# Local trampling disturbance effects on alpine plant populations and communities: Negative implications for climate change vulnerability

**DOI:** 10.1002/ece3.4276

**Published:** 2018-07-16

**Authors:** Nathalie Isabelle Chardon, Sonja Wipf, Christian Rixen, Annabarbara Beilstein, Daniel Forest Doak

**Affiliations:** ^1^ Environmental Studies Program University of Colorado Boulder USA; ^2^ Mountain Ecosystems WSL Institute for Snow and Avalanche Research SLF Davos Switzerland; ^3^ Department of Environmental Systems Science ETH Zurich Zurich Switzerland

**Keywords:** alpine, climate change, disturbance, facilitation, *Silene acaulis*, Switzerland

## Abstract

Global change is modifying species communities from local to landscape scales, with alterations in the abiotic and biotic determinants of geographic range limits causing species range shifts along both latitudinal and elevational gradients. An important but often overlooked component of global change is the effect of anthropogenic disturbance, and how it interacts with the effects of climate to affect both species and communities, as well as interspecies interactions, such as facilitation and competition. We examined the effects of frequent human trampling disturbances on alpine plant communities in Switzerland, focusing on the elevational range of the widely distributed cushion plant *Silene acaulis* and the interactions of this facilitator species with other plants. Examining size distributions and densities, we found that disturbance appears to favor individual *Silene* growth at middle elevations. However, it has negative effects at the population level, as evidenced by a reduction in population density and reproductive indices. Disturbance synergistically interacts with the effects of elevation to reduce species richness at low and high elevations, an effect not mitigated by *Silene*. In fact, we find predominantly competitive interactions, both by *Silene* on its hosted and neighboring species and by neighboring (but not hosted) species on *Silene*. Our results indicate that disturbance can be beneficial for *Silene* individual performance, potentially through changes in its neighboring species community. However, possible reduced recruitment in disturbed areas could eventually lead to population declines. While other studies have shown that light to moderate disturbances can maintain high species diversity, our results emphasize that heavier disturbance reduces species richness, diversity, as well as percent cover, and adversely affects cushion plants and that these effects are not substantially reduced by plant–plant interactions. Heavily disturbed alpine systems could therefore be at greater risk for upward encroachment of lower elevation species in a warming world.

## 1 INTRODUCTION

Expected shifts in species geographic distributions in response to climate change have spurred numerous studies to determine which abiotic (e.g., climatic) and biotic (e.g., competitive and facilitative) processes determine range limits and affect population performance (Sexton, McIntyre, Angert, & Rice, 2009). One topic of these studies is understanding the effects of disturbance regimes and potential shifts in disturbance patterns with climate change. However, despite their significant potential to alter competitive balances or override climatic effects, the role of localized anthropogenic factors (e.g., site‐specific disturbance regimes) in shaping range limits, including their interactions with broader climate changes, remains surprisingly understudied (Turner, 2010). To predict how populations at range limits will respond in an era of climate warming, it is therefore crucial to understand how the cumulative effects of local disturbance, climate, and species interactions influence population parameters. This is especially relevant in systems where declining performance of threatened trailing edge (i.e., warmer climatic edge) populations could cause range contractions, such as for species that occur across substantial elevational gradients. For these species, effects of local disturbance would be expected to interact with the known negative effects of encroachment of lower elevational, more competitive, species (Alexander, Diez, & Levine, 2015) in ways that could either stabilize lower range limits or, conversely, cause them to fail such that the entire range shifts upward in response to climate change.

Trailing edge populations are particularly threatened by the impacts of climate change, with possible mechanisms including increasingly warm temperatures and encroachment by formerly restricted lower latitude or lower elevation species (Parmesan, 2006). In mountain systems, where lower and upper limits are often believed to be set by biotic and abiotic factors, respectively (e.g., Ettinger, Ford, & HilleRisLambers, 2011), such encroachment can result in lower elevational range contractions (e.g., Kopp & Cleland, 2015). This pattern in turn relies on lower elevation species having higher competitive abilities than those characteristically living at higher elevations. If this pattern holds, we would expect that alpine species would be unable to maintain their lower elevational limits in the face of increased competition resulting from climate change. However, this set of processes may be moderated by multiple other factors, including local disturbance. In particular, it is unclear how the biotic interactions that influence species range limits will shift with climate change, and particularly how the strength of these interactions will be altered by disturbances.

Disturbance has long been recognized as an important driver of ecosystem dynamics (e.g., Connell, 1978), and high‐intensity disturbance can exert significant organismal damage (Barros & Pickering, 2015). Disturbance interacts strongly with multiple biotic processes (see Pickett & White, 1985 for review, pp. 287–316) and can even override the effects of climate (Franklin, Serra‐Diaz, Syphard, & Regan, 2016). It can determine distributional patterns, such as in the cases of recurring fires (Sousa, 1984) or through changes in landscape patch structure (Pickett & White, 1985; p. 309). Anthropogenic disturbances can broaden the range in which non‐native species can grow (Lembrechts et al., 2017), favor invasive species richness (Sandoya, Pauchard, & Cavieres, 2017), and cause distributional shifts in invasive species (e.g., McKenzie, Yoshida, & Unsworth, 2014). Given its influential role in invasive species range expansion, it is therefore surprising that disturbance is often left out of most studies of native species range limits. This limits a comprehensive understanding of how disturbance affects range‐limiting mechanisms, or how such interactive effects will respond to global climate change. We would expect that disturbance will influence range limits, in particular for species such as many alpine plant species that are limited by competition at their lower elevational range limit (Choler, Michalet, & Callaway, 2001). In abiotically benign areas, where facilitative effects of alpine species are marginal (Callaway et al., 2002), competitive interactions dominate ecosystem processes. If disturbance reduces competitive interactions by reducing the density of dominant competitors, we might expect alpine species to exhibit enhanced performance or density with moderate disturbance, possibly stabilizing lower range limits in the face of climate change. Of course, this effect will only occur if disturbance is not so intense as to exert strong direct negative effects on alpine species themselves.

While disturbance may reduce competitive interactions at lower elevational limits, we would expect quite different effects at higher elevations. The facilitative effects of cushion plants, in particular, is generally believed to increase along elevational gradients, as they provide the necessary microhabitat for hosted species living within the cushions at high elevations characterized by increased abiotic stress (Callaway et al., 2002). These nurse plants may therefore play an important role in maintaining high species diversity around the globe (Butterfield et al., 2013). However, studies suggesting that cushion plants augment overall species richness (e.g., Cavieres, Hernandez‐Fuentes, Sierra‐Almeida, & Kikvidze, 2016) have been countered by other work showing that cushion species actually host less‐diverse communities than surrounding areas (e.g., Dvorsky et al., 2013). Considering that disturbance is a form of abiotic stress, we expect alpine facilitator species to host increased species not only because these facilitators provide a more sheltered microhabitat, but also because of the reduced resistance of facilitator species to other species. This is especially likely at higher elevations, where abiotic stress is known to play a large part in determining ecological processes.

To the extent that disturbance alters community interactions, such as facilitation and competition, it could have strong indirect effects on community assembly and species diversity. There is evidence that disturbance can affect facilitative and competitive interactions, such as reducing facilitator species’ reproductive output and increasing hosted species presence (Michalet et al., 2011). On the other hand, facilitative interactions can break down with high levels of abiotic stress (for review see Michalet & Pugnaire, 2016). Not only do we lack a clear picture of which environmental factors influence these interactions, but we also do not have a comprehensive understanding of the role that disturbance plays on species interactions along biotic and abiotic stress gradients, and how this influences species range limits.

**Figure 1:**
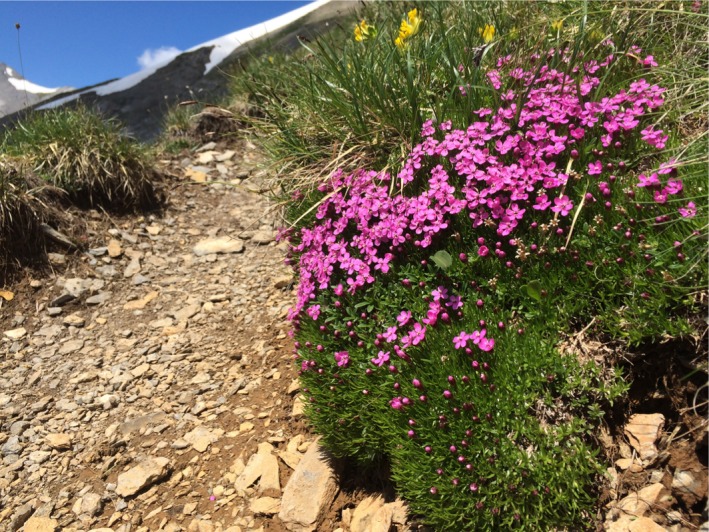
**Study species**. *Silene acaulis* is a facilitative alpine cushion plant found throughout the Northern Hemisphere

In order to address the question of how disturbance can influence range limits, we focused on the biotic to abiotic gradient often present along elevational gradients in alpine ecosystems. While disturbances can be short‐ to long‐term and natural or anthropogenic in origin, we studied the margins of human‐made trails, which represent frequent, relatively high‐intensity disturbances that are similar to livestock trails. Livestock trails are, however, more damaging, not only because livestock exert more pressure on the ground, but also because livestock herds create multiple trails (Barros et al., 2015; Cole & Spildie, 1998; Pickering, Hill, Newsome, & Leung, 2010). We specifically examined trail‐side and off‐trail plant communities in a system known to exhibit facilitative and competitive interactions along elevational gradients in the Swiss Alps. To assess the net effects of disturbance on such interactions, we quantified performance indicators of the well‐studied facilitative common alpine cushion plant species, *Silene acaulis* (L.) Jaq. (Caryophyllaceae; Figure 1), and quantified community measures of its inside (plants growing within cushions) and neighboring (plants growing next to cushions) species. Collecting data on the responses of a facilitative species as well as its inside and neighboring species allowed us to better understand (a) how disturbance influences survival, growth, and reproduction indicators of this individual facilitative species and (b) how this community and its interactions are altered by disturbance. Specifically, we tested the following hypotheses:

(a) At low elevations, presumably characterized by low abiotic stress and increased competition, disturbance will largely benefit cushion plant growth (as indicated by size of plants). At abiotically stressful high elevations, disturbance will have net negative effects. (b) Disturbance may, however, have a negative effect on population density at all elevations, possibly due to low establishment and survival of younger plants.
Higher abundance of species inside disturbed cushions will have negative effects on cushion plant reproduction at all elevations.
Facilitation by cushion plants will be stronger and more important in maintaining species diversity in disturbed areas, an effect amplified at higher elevations.

To test these hypotheses, we measured *Silene acaulis* (henceforth, *Silene*) populations and species community structure along elevational range locations at sites frequently disturbed by human trampling (i.e., hiker trails) vs. relatively undisturbed (i.e., off‐trail) areas in southeast Switzerland. We additionally measured two soil parameters (soil organic matter and soil water content) to understand how disturbance alters habitat conditions. *Silene* is an ideal model species for this work, as it is a common circumboreal alpine plant with important facilitative effects on other vegetation (Butterfield et al., 2013). Its widespread distribution and facilitative effects make it an important alpine species across the Northern Hemisphere, and drivers of change to its populations, such as disturbance, need to be examined in order to improve our understanding of how to maintain alpine biodiversity in the face of impacts by multiple interactions.

## 2 MATERIALS AND METHODS

### 2.1 Sites

**Figure 2:**
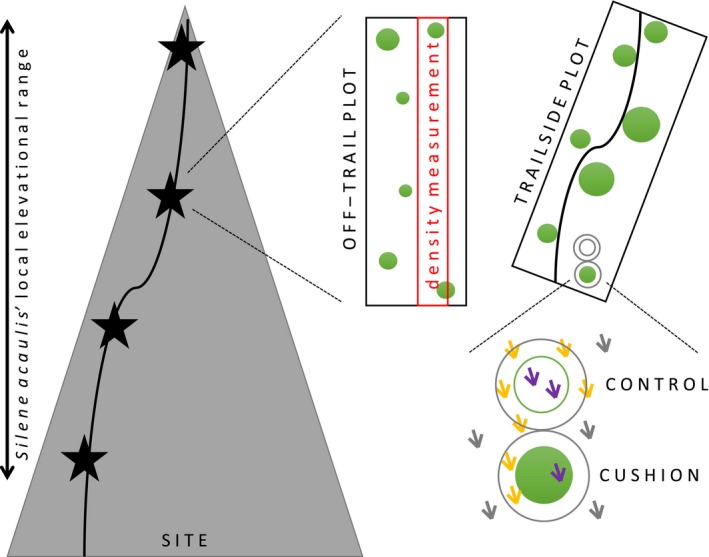
**Sampling design**. The black curved line represents a hiking trail at a SITE, and stars indicate sampling locations along *Silene acaulis*’ local elevational range. Black rectangles delineate each PLOT, and the smaller, inner red rectangle within the plot was used to calculate population density at both off‐trail and trail‐side plots. Green circles are cushion plants (*n* = 30 per plot), and each plot had randomly chosen focal cushions for CUSHION/CONTROL pairs (*n* = 5 per plot). Gray circles represent the 5 cm sampling belt outside cushion and control (inner green circle) area. Cartoon plants are other vegetation, with purple cartoons measured as inside species and orange cartoons as neighboring species. Gray cartoons were not measured as they were outside the sampling area. See text for additional details

We established three sampling sites located along popular alpine hiking trails on two summits and one mountain pass (Piz Beverin, Haldensteiner Calanda, Fallerfurgga) within the canton of Grisons in southeastern Switzerland. We chose the summits using known occurrence locations (InfoFlora 2016) to ensure that sampling sites span *Silene*'s elevational range. At four evenly spaced elevations (i.e., elevational levels) encompassing *Silene*'s local (i.e., within site) elevational range, we sampled disturbed (trail‐side) and paired undisturbed (off‐trail) plots with a standard width (1 m for trail‐side plots and 5 m for off‐trail plots) and variable length (mean size = 16 m^2^) between June and August 2016. We defined plots as the area including the first 30 *Silene* individuals we encountered at each elevational level. For trail‐side plots, we marked the first 30 *Silene* individuals within 0.5 m on either side of the trail while walking uphill. For off‐trail plots, we walked at least 10 m away from the trail to find an undisturbed (i.e., no hiker or livestock trail) area of similar topography as the trail, and marked the first 30 *Silene* individuals while walking uphill, back and forth in a 5 m width (Figure 2).

Plots span an elevational range of 1,950–2,680 m, are characterized by a continental alpine climate, and have a bedrock type predominantly classified as biogenic sedimentary rock (Federal Office of Topography, 2016). The summer growing season (June, July, August) has a mean monthly temperature of 5°C and mean monthly precipitation of 180 mm, and annual precipitation is 1,411 mm (1981‐2010 at Weissfluhjoch Weather Station; Federal Office of Meteorology and Climatology MeteoSwiss 2017). These sites have been moderately grazed by livestock (mainly cattle and sheep) for centuries, and the trails we sampled have been used as mountain passages for over a century. These sites are currently still used by livestock, with higher use at lower elevations, and livestock use is similar between sites. As evidenced by low dung counts at all sites (*pers. observation*), grazing intensity is low. Hikers utilize these popular trails to hike to the summit or nearest pass, with similar hiker numbers at all elevations.

### 2.2 Field measurements: cushion plants

At each plot, we measured the size (i.e., cushion area, following the methods of Doak & Morris, 2010) of all 30 *Silene* individuals regardless of cushion size for data to test Hypothesis 1a. To estimate population density within each plot in order test Hypothesis 1b, we delineated an area of 0.5 m (to achieve a standard width within trail‐side and off‐trail plots) by the maximum length of the plot and recorded which *Silene* individuals we found within it. We picked this area to be the 0.5 m width within the plot that had the highest density of plants, and fit this area to trail curvature for trail‐side plots (Figure 2). Of the 30 individuals measured per plot, we randomly picked five individuals (henceforth, “focal plants”) for additional measurements of either flower or fruit number (depending on individual plant phenology at the time of censoring) and sex (hermaphrodite or female) in order to test Hypothesis 2.

### 2.3 Field measurements: community effects and species interactions

For each of the five focal plants in each plot, we established a control area of the same size but without any *Silene* cushion (methodically selected within 0.1–0.5 m of the focal plant with similar slope, aspect, and microtopography), using wire loops to maintain size of cushion area (following methods of Butterfield et al., 2013). We identified the identity and percent cover of other plant species growing inside each cushion and control area (i.e., inside species), as well as within 5 cm of the cushion edge and control edge (i.e., neighboring species) for data to test Hypothesis 3. Our sampling protocol yielded 5 cushion/control pairs per disturbance type by elevation and 30–40 pairs per site, totaling 100 pairs.

To characterize soils from cushions and controls, we extracted soil samples at 4 cm depth using a spoon of approximately 20 cm^3^ at three cushion/control pairs per plot. We placed each soil sample in a plastic bag in the field. We determined soil water content (% SWC) by weighing the soil samples before and after drying them >48 hr at 60°C. We determined soil organic matter content (% SOM) of sieved soil samples (at 2 mm mesh size) by the loss on ignition method: 2 subsamples of 2 g dry soil per sample burned at 410°C for 40 hr (following the methods of Schöb, Butterfield, & Pugnaire, 2012), and weighed again after cooling. We averaged the values of the two samples for our measure of % SOM. At each plot, we measured microhabitat temperature over 1 year with temperature loggers (Maxim Integrated iButtons, CA, USA) buried at 2 cm depth under one of the focal cushions and its corresponding control.

### 2.4 Statistical analyses: cushion plants

To test whether disturbance largely benefits cushion plant growth at low elevations and has a net negative effect at high elevations (Hypothesis 1a), we first examined size distribution differences between disturbed and undisturbed *Silene* individuals with a Kolmogorov–Smirnov test. Second, to further test Hypothesis 1a and to test whether population density is reduced by disturbance at all elevations (Hypothesis 1b), we quantified the effects of disturbance and elevation on the plot‐level densities and on individual size of *Silene* plants (Supporting Information Table A1a in Appendix S1) using two separate sets of linear mixed models (LMMs; see below for details). Third, to test if higher abundance of species inside disturbed cushions has a negative effect on reproduction across all elevations (Hypothesis 2), we examined the effects of disturbance, elevation, and several community indices (Supporting Information Table A1b in Appendix S1) on *Silene* reproduction indicators (fruit density, relative reproduction) by fitting another set of LMMs. We fit a separate model set using either inside or neighboring community measures, in order to understand effect differences from species growing within cushions (inside species) compared to those growing adjacent (neighboring species). Lastly, we tested the effects of disturbance, level, SOM, and SWC on *Silene* cushion size and reproduction indicators, to understand how disturbance‐mediated changes in habitat are important.

In each set of LMMs, we fit a series of alternative models for each dependent variable with differing combinations of main effects (Supporting Information Table B1 in Appendix S2), with all models including a random intercept and a random site effect. We included the explanatory variable of elevational level in all model sets, as this metric had much higher overall predictive power than absolute elevation, elevation above lowest local *Silene* occurrence, average June temperature, or average July temperature. As demonstrated by our microhabitat temperature data, elevational level is a fairly good predictor of average June temperature (conditional *r* ^2^ = 0.50, *p*‐values_levels_ < 0.05). We identified the most parsimonious model in each model set using AICc. To identify meaningful explanatory variables within model sets with multiple models within 2 AICc, we computed AICc weighted average ratios of t values (Cade, 2015). We performed all analyses with the R (Version 3.4.1) programming language (R Core Team 2017). We fit LMMs in the “lme4” package (Bates, Maechler, Bolker, & Walker, 2015), and calculated additional outputs using the “AICcmodavg” (Mazerolle, 2016) and “MuMIn” (Bartoń, 2016) packages.

We calculated two reproduction indices, fruit density and relative reproductive success. Due to differences in sampling times and phenology, some plants were in flower and others in fruit when sampled. We therefore converted flower to fruit number for plants of each sex using relationships from 628 individual *Silene* plants from Colorado, USA (D. F. Doak, W. F. Morris, and M. L. Peterson, unpublished data; no comparable local data were available). These data show strong and significant correlations between flower number and seed‐bearing fruits within the same growing season (females: *p*‐value < 0.001, *r* ^2^ = 0.79; hermaphrodites: *p*‐value < 0.001, *r* ^2^ = 0.70; Supporting Information Figure A1a in Appendix S1).

We used fruit density (number of fruits/cushion size) as a broad measure of reproductive output. We also quantified relative reproductive output through several steps to arrive at a size‐ and sex‐independent measure of relative reproduction. We first regressed fruit number on cushion area for each sex, and then as an index of relative reproductive success divided each plant's residual by the predicted value for its sex and size. Values greater than one indicate high reproductive rate while those below one show less than expected production. We also tested whether fruit production correlates with other aspects of individual performance by regressing relative reproductive rate on relative growth rate for the Colorado data set, and found that the two values are weakly correlated (*r* ^2^ = 0.14; Supporting Information Figure A1b in Appendix S1). Neither relative growth nor relative fruit production is significantly dependent on cushion size (Supporting Information Figure A1c, d in Appendix S1).

### 2.5 Statistical analyses: community effects

We quantified communities in several ways. First, we used direct data on the non‐*Silene* plants in each cushion or control area to determine absolute species richness, Shannon diversity (“vegan” package; Oksanen et al., 2017), percent cover of non‐*Silene* plants, and community competitiveness. We derived species competitive values from species indicator values assigned to each species in Switzerland (Landolt et al., 2010). Each species has a value indicating its position on Grime's Triangle, such that most competitive species are coded as “ccc,” most ruderal as “rrr,” and most stress‐tolerant as “sss,” with any combination of three letters possible. We assigned each species a competitive value from 0 to 3 according to how many “c”s its three‐letter code contained. For each sampling unit (i.e., individual cushion, control, or their respective neighboring rings), we calculated the species average competitive value.

To test if facilitation by disturbed cushion plants is stronger and more important in maintaining species diversity at higher sites (Hypothesis 3), we examined the effects of disturbance, elevation, and *Silene* presence on community characteristics with a set of LMMs separately for species richness, Shannon diversity, and percent vegetation cover (Supporting Information Table A2 in Appendix S1). These models include different combinations of elevation, disturbance, *Silene* presence, and sample size area, with sample size never tested without added effect of cushion presence (Supporting Information Table B2 in Appendix S2). To improve model stability, we centered and scaled sampling area. Model details are as described above, with a nested random effect of site and cushion‐control pair. To examine how community competitiveness is influenced by disturbance, elevation, and cushion presence, we fit LMMs with these all combinations of these three parameters separately on inside and neighboring average community competitive index (Supporting Information Table B3 in Appendix S2). Model details are as described above, with a nested random effect of site.

In order to understand how soil parameters influence species richness, diversity, and percent cover, we removed cushion presence and included SOM and SWC in our inside species LMMs (Supporting Information Tables A2 and B2 in Appendices S1 and S2, respectively). To improve model stability, we centered and scaled SOM and SWC. We did not include these soil parameters in our first model set, as this dataset has a smaller sample size. To then understand how cushion presence, disturbance, and elevation influence SOM and SWC, we tested these effects with LMMs (Supporting Information Tables A3 and B4 in Appendices S1 and S2, respectively). Since soil samples were taken underneath cushions and their respective controls, and not separately for neighboring environments, we could only test for effects on inside species. Model details are as described above, with a nested random effect of site and cushion‐control pair.

### 2.6 Statistical analyses: species interactions

In order to account for the species differences observed between each focal plant and its associated control area, we calculated two separate indices. The Bray–Curtis dissimilarity index is a measure of compositional dissimilarity between two sites (Bray & Curtis, 1957), which we calculated using the “vegan” package (Oksanen et al., 2017). We calculated separate dissimilarities between a focal plant and its control (i.e., inside species), and between the 5 cm neighboring ring around a focal plant and the replicated ring around its control (i.e., neighboring species). The relative interaction index (RII; Armas, Ordiales, & Pugnaire, 2004) is a measure of interaction intensity between plants, with positive values indicating facilitation and negative values competition. We calculated a RII between the cushion vs. control inside species and the cushion vs. control neighboring species as follows: RII = (*N*_cushion_–*N*_control_)/(*N*_cushion_ + *N*_control_), where N is species richness (RII_s_), species diversity (RII_shan_), or total percent cover (RII_cov_).

Following many alpine facilitation studies and as part of our test of Hypothesis 3, we tested for effects on RII and Bray–Curtis dissimilarity values with LMMs. These models include disturbance and elevation as fixed effects, and site as a random effect (Supporting Information Tables A4 and B5 in Appendices S1 and S2, respectively). All models were structured as described in the previous section, and we tested the effects on inside and neighboring species separately.

## 3 RESULTS

### 3.1 Cushion plants

**Figure 3:**
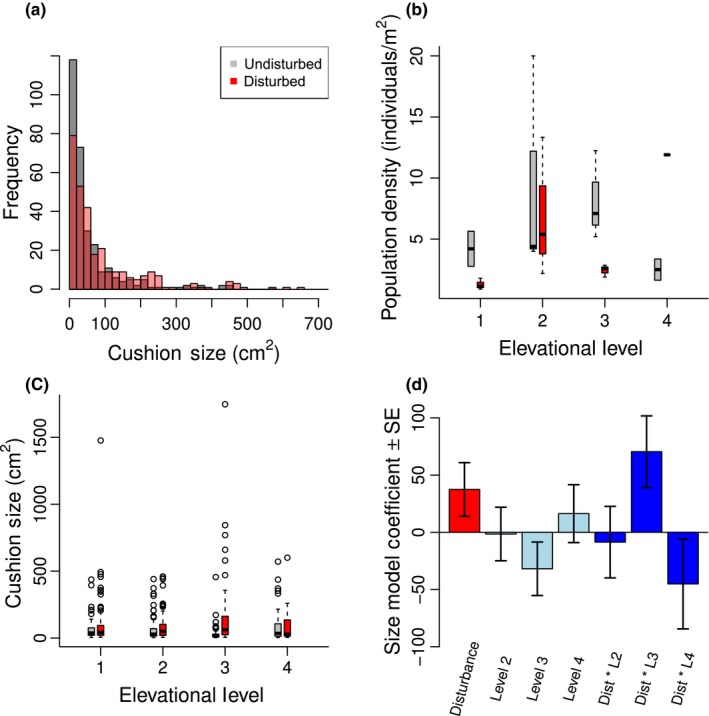
**Disturbance effects on** ***Silene acaulis.*** (a) Disturbed sites have smaller numbers of small *Silene acaulis* individuals, and increased numbers of larger individuals (12 largest sizes removed to improve figure clarity). Population density (b) is highest at the center of the species range (levels 2 and 3), with no effect of disturbance in the most parsimonious model but a moderate negative disturbance effect over the full model set (colors as in (a)). Disturbance increases *Silene acaulis* mean cushion sizes (c, colors as in (a)). The best supported model for cushion size (d) includes a positive disturbance effect, a negative unimodal elevation effect, and a significant disturbance by elevation effect. This suggests that although disturbance benefits cushion growth at middle elevations (level 3), it greatly inhibits it at the upper elevational range limit (level 4). Contrasting colors merely differentiate parameters

We predicted that disturbance will benefit cushion plant growth at low elevations and have a net negative effect at high elevations (Hypothesis 1a), and have a negative effect on population density at all elevations (Hypothesis 1b). We found that disturbed and undisturbed *Silene* individuals have significantly different sizes (Figure 3a) as well as different size distributions (Supporting Information Figure A2 in Appendix S1), with disturbed areas having much larger maximum plant sizes and undisturbed areas having more small individuals. While these results suggest benefits for plant growth from disturbance, our models indicate a possible role of disturbance in decreasing population density. Although the most parsimonious model for *Silene* population density indicates that density is highest in the middle of *Silene*'s elevational range and does not include a disturbance effect (Table 1A, Figure 3b), the full model set indicates a moderate negative effect of disturbance on population density (AICc weighted average ratio of *t* value = 0.78). *Silene* mean cushion sizes are increased by disturbance (Table 1A; Figure 3c), implying older age of plants, faster growth rates, or both. This relatively weak effect is largest in the middle of the species’ elevational range (level 3), with a significant disturbance by elevation interaction supported by model selection (Figure 3d). Compared to undisturbed cushions, disturbed cushions were on average 128% larger at middle elevations (level 3) but only 30% larger at range edges (levels 1, 2 and 4).

**Table 1:** Results of most parsimonious models testing the effects of (A) disturbance and elevational level on cushion size and population density and (B) disturbance, level, and species community indices on reproduction indicators. Response variables subscripts indicate if tested community indices correspond to inside or neighboring species. Light green colors differentiate response variables tested using the same dataset; black differentiates different datasets. Interactions (Int(s)) are listed without the corresponding estimates. Elevational level and disturbance are factor variables, with 4 and 2 levels, respectively. Level coefficient values are hence summarized as follows: (+) positive trend, (−) negative trend, or unimodal with a maximum (+) or minimum (−) at levels 2 or 3. All models with Δ AICc values of less than 2 are shown for each response variable with marginal (marg) *r* ^2^ and conditional (cond) *r* ^2^ listed, and significant *p*‐values (<0.001***, <0.01**, <0.05*) shown above the first listed model within each section. *p*‐Values for level indicate that at least one level was significant at <0.05. The full list of models tested and their AICc weights are shown in Supporting Information Table B1 in Appendix S2

(A)							
Response variable	Intercept	Disturbance	Level	Int(s)	marg *r* ^2^	cond *r* ^2^	Δ AICc
				*			
Silene size	62.48	37.44	Unimodal (−)	Dist × level	0.05	0.06	0.00
							
Population density	2.45		Unimodal (+)		0.16	0.16	0.00
Population density	3.66	−1.98	Unimodal (+)		0.19	0.19	0.64
Population density	4.86	−3.58	Unimodal (+)	Dist × level	0.35	0.44	0.79

(B)											
Response variable	Intercept	Disturbance	Level	Richness	Diversity	% Veg cover	Competition	Int(s)	marg *r* ^2^	cond *r* ^2^	Δ AICc
Fruits per area_inside_	0.13								0.00	<0.01	0.00
Fruits per area_inside_	0.46						−0.28		0.01	0.01	0.68
											
Relative reproduction_inside_	−0.20		Unimodal (−)				0.26	Level × comp	0.12	0.19	0.00
Relative reproduction_inside_	−4.13	1.11	+				3.21	Level × comp	0.17	0.31	1.49
											
		**			***			**			
Fruits per area_neighboring_	2.32	−2.23			−1.07			Dist × diversity	0.15	0.16	0.00
											
Relative reproduction_neighboring_	1.31		Unimodal (−)				−0.94	Level × comp	0.25	0.42	0.00

**Figure 4:**
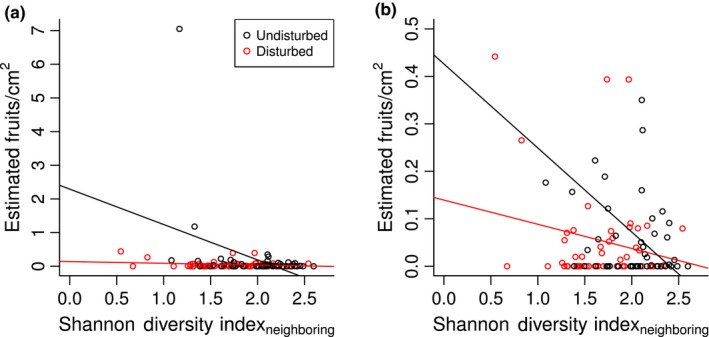
**Disturbance effects on** ***Silene acaulis*** **reproduction**. Fruit density is negatively affected by both neighboring species diversity and disturbance, with a significant disturbance by diversity interaction that implies the negative effect of disturbance overrides those of diversity. Linear regression lines based on only the fixed effect of Shannon diversity index and shown separately for disturbed and undisturbed cushions, where undisturbed cushions are significantly negatively affected by diversity (a) without and (b) with two outliers removed (colors as in (a)) (respective *p*‐values = 0.007, 0.018). Note the different *y*‐axes scales. Points jittered for clarity

We further predicted that higher abundance of species inside disturbed cushions will have negative effects on reproduction at all elevations (Hypothesis 2). We found that *Silene* reproduction is best explained by models with neighboring, but not inside, community indices (Table 1B). Both disturbance and neighboring species diversity significantly reduce fruit density (although not neighboring species abundance, as measured by percent cover), with a significant disturbance by diversity interaction effect (Figure 4). Contrary to our expectations, fruit density is not influenced by any inside species measures, and neither inside nor neighboring species measures have a significant effect on relative reproduction.

In model sets testing the effects of SOM and SWC, which replaced species community parameters, we found that higher values in both soil parameters relate to decreased *Silene* reproductive measures. SWC decreases fruit density and SOM moderately decreases relative reproduction, with a negative effect of disturbance on fruit density (Supporting Information Table A5a in Appendix S1). In these models, fruit density is highest at both upper and lower elevational range edges, and relative reproduction decreases with elevation. The best model for cushion size has no significant explanatory variables.

### 3.2 Community effects: inside species

**Figure 5:**
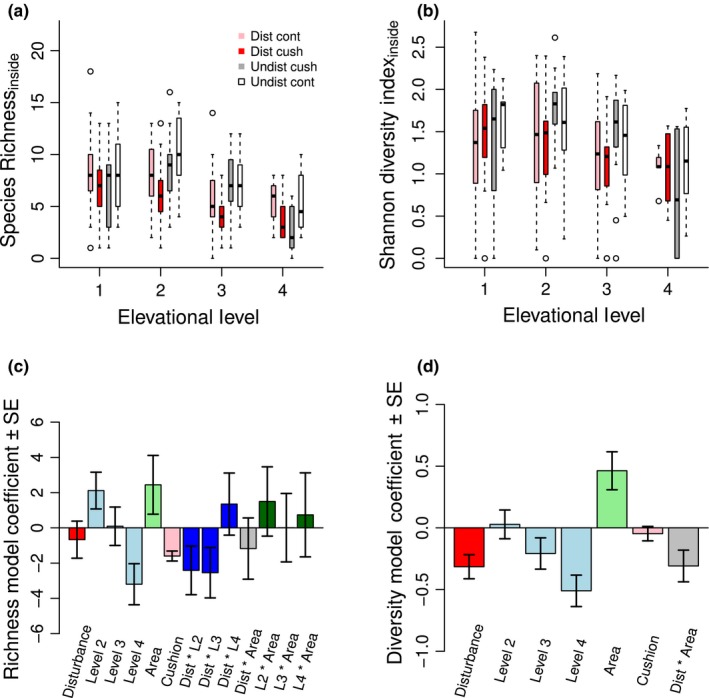
**Inside species community**. Disturbance reduces inside species richness (a) and diversity (b), which both decrease with cushion presence (colors for b as in (a)). Legend abbreviations are as follows: dist = disturbed, undist = undisturbed, cush = cushion, cont = control. The best supported model for species richness (c) highlights the importance of interactions between disturbance and elevation, which synergistically interact to decrease richness at middle elevations (levels 2 and 3). The most parsimonious model for species diversity (d) suggests that the interaction between disturbance and cushion area cancels out the positive effect of area. Contrasting colors merely differentiate parameters

We predicted that facilitation by cushion plants will be stronger and more important in maintaining species diversity in disturbed areas, an effect amplified at higher elevations (Hypothesis 3). However, we did not find an amplified facilitative effect on inside species by *Silene* cushions in disturbed areas, or support for any other interaction between cushion presence and disturbance (Table 2). In contrast to findings of some previous studies, cushion presence has a significant negative effect on species richness (Figure 5a,c), and a moderate negative effect on both Shannon diversity (Figure 5b,d) and percent vegetation cover (Supporting Information Figure A3a,c in Appendix S1).

**Table 2:** Results of most parsimonious models testing the effects of disturbance, elevational level, *Silene acaulis* cushion presence, and sampling area on species community indices. Light green colors differentiate response variables tested using the same dataset, black differentiates different datasets. Interactions (Int(s)) are listed without the corresponding estimates. Elevational level and disturbance are factor variables, with 4 and 2 levels, respectively. Level coefficient values are hence summarized as follows: (+) positive trend, (−) negative trend, or unimodal with a maximum (+) or minimum (−) at levels 2 or 3. All models with Δ AICc values of less than 2 are shown for each response variable with marginal (marg) *r* ^2^ and conditional (cond) *r* ^2^ listed, and significant *p*‐values (<0.001***, <0.01**, <0.05*) shown above the first listed model within each section. *p*‐Values for level indicate that at least one level was significant at <0.05. The full list of models tested and their AICc weights are shown in Supporting Information Table B2 in Appendix S2

Response variable	Intercept	Disturbance	Level	Cushion	Area	Int(s)	marg *r* ^2^	cond *r* ^2^	Δ AICc
			*	***					
Species richness_inside_	8.64	−0.67	Unimodal (+)	−1.60	2.44	Dist × level	0.44	0.72	0.00
									
		**	*		***	*			
Shannon diversity_inside_	1.69	−0.31	Unimodal (+)	−0.05	0.46	Dist × area	0.24	0.57	0.00
Shannon diversity_inside_	1.64	−0.27	Unimodal (+)	−0.05	0.21		0.21	0.57	1.16
Shannon diversity_inside_	1.54	−0.27		−0.05	0.48	Dist × area	0.16	0.56	1.50
									
			*	***					
% Vegetation cover_inside_	62.32	0.78	Unimodal (+)	−37.42	3.09	Dist × level × area	0.53	0.59	0.00
									
			*						
Species richness_neighboring_	12.03	−0.71	Unimodal (+)	−0.42	−0.01	Dist × level × area	0.37	0.75	0.00
									
Shannon diversity_neighboring_	2.08	−0.27	—				0.21	0.58	0.00
									
% Vegetation cover_neighboring_	64.85	1.89	Unimodal (+)	−1.08	−15.99	Dist × level × area	0.41	0.79	0.00

As expected, we found that disturbance exerts an overall negative effect on both the species richness and diversity of inside species (Table 2). Although disturbance has an overall net positive effect on percent vegetation, visual interpretation of the three‐way interaction with elevational level and area demonstrates that disturbance effects are weak at low and high elevations but strongly negative at middle elevations (for additional analysis see Supporting Information Table B6 in Appendix S2). All three community measures of inside species are highest at middle elevations and increase with sampling area. The interaction effect of area for all three community measures is likely due to larger cushion sizes (and therefore larger sampling areas) in disturbed areas, and varying cushion sizes across elevations.

We found that inside community competitiveness is significantly lower at higher elevations, with no effect of cushion presence and disturbance (Supporting Information Figure A4a, Table A6 in Appendix S1). This pattern is most likely not driven by certain highly competitive individual species alone, but rather by the average competitive index values found at overall median species richness (Supporting Information Figure A5a in Appendix S1).

After including the sampled soil parameters as predictor variables in our models, we found that higher values of SWC are related to higher inside species community richness and percent vegetation cover, but SWC has no effect on diversity (Supporting Information Table A5b in Appendix S1). Higher SOM values decrease species richness and percent vegetation cover, and SOM also has no effects on diversity. Both species richness and percent vegetation cover are increased with disturbance and are highest at middle elevations, with a 4‐way interaction (SOM × SWC × disturbance × elevation) present for both. These soil parameters, in turn, are negatively influenced by disturbance, both peak at middle elevations, and are positively affected by *Silene* presence (Supporting Information Table A7 in Appendix S1).

### 3.3 Community effects: neighboring species

**Figure 6:**
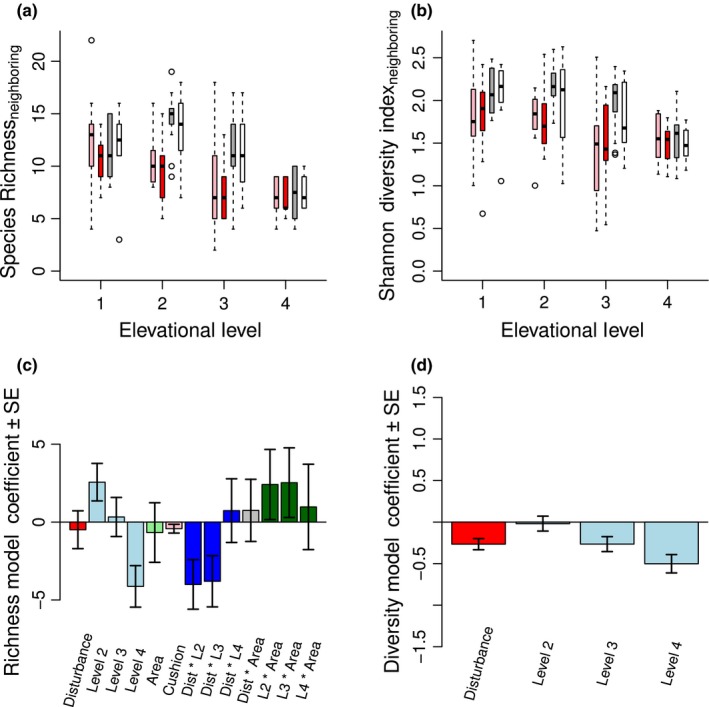
**Neighboring species community**. Disturbance reduces neighboring species richness (a) and diversity (b) (colors as in Figure 5a), with an additional negative effect of cushion presence and area on richness. The best supported model for species richness (c) highlights the importance of interactions between disturbance and elevation, whose effects synergistically interact to decrease richness at middle elevations (levels 2 and 3; see also Supporting Information Table B6 in Appendix S2), an effect partly mitigated by the interaction between elevation and area. The most parsimonious model for species diversity (d) indicates a negative effect of disturbance and level, with no effect of cushion. Contrasting colors merely differentiate parameters

As for inside species, we did not find evidence that facilitation by *Silene* cushions on neighboring species increases with disturbance (Table 2). Surprisingly, *Silene* presence has a moderate negative effect on species richness (Figure 6a,c) and percent vegetation cover (Supporting Information Figure A3b,d in Appendix S1). As expected, we found an overall moderate negative effect of disturbance on both species richness and Shannon diversity, with the effects of disturbance on diversity most pronounced at middle elevations (Figure 6b,d). As for inside communities, neighboring species richness peaks at middle elevations, and Shannon diversity decreases with elevation. Both neighboring species richness and percent vegetation cover decrease with sampling area. Disturbance has an overall positive effect on vegetation cover, but as seen through visual interpretation of the three‐way interaction with elevational level and area, disturbance exerts weak effects at low and high elevations with strong negative effects at middle elevations (for additional analysis see Supporting Information Table B6 in Appendix S2).

We found that neighboring species community competitiveness is highest at middle elevations, with no influence by disturbance (Supporting Information Figure A4b in Appendix S1). As with inside species, we suspect that this pattern is driven by sampling areas that exhibit median species richness (Supporting Information Figure A5b in Appendix S1).

### 3.4 Species interactions

Contrary to our third hypothesis, we observed neither an increase in facilitation with disturbance nor an overall facilitative effect by *Silene* on neither inside nor neighboring species. Our data show more negative RII values than expected (Supporting Information Figures A6, A7 in Appendix S1), indicating net competition within cushions and between cushions and neighboring species. We found no support of a disturbance effect on RII_cov_, RII_shan_, RII_veg_, and the Bray–Curtis dissimilarity index nor along our sampled elevational gradient (Table 3).

**Table 3:** Results of most parsimonious models testing the effects of disturbance and elevational level on relative interaction indices (RII) and Bray–Curtis dissimilarity indices (calculated between cushions and corresponding controls). Inside: species inside cushions compared to species inside control; neighboring: cushion neighbors compared to control neighbors. Light green colors differentiate response variables tested using the same dataset; black differentiates different datasets. Interactions (Int(s)) are listed without the corresponding estimates. Elevational level and disturbance are factor variables, with 4 and 2 levels, respectively. Level coefficient values are hence summarized as follows: (+) positive trend, (−) negative trend, or unimodal with a maximum (+) or minimum (−) at levels 2 or 3. All models with Δ AICc values of less than 2 are shown for each response variable with marginal (marg) *r* ^2^ and conditional (cond) *r* ^2^ listed, and significant *p*‐values (<0.001***, <0.01**, <0.05*) shown above the first listed model within each section. *p*‐Values for level indicate that at least one level was significant at <0.05. The full list of models tested is shown in Supporting Information Table B5 in Appendix S2

Response variable	Intercept	Disturbance	Level	Int(s)	marg *r* ^2^	cond *r* ^2^	Δ AICc
RII: Species richness_inside_	−0.13				0.00	0.10	0.00
							
RII: Shannon diversity_inside_	−0.04				0.00	0.17	0.00
							
RII: % Vegetation cover_inside_	−0.42				0.00	0.02	0.00
							
Bray–Curtis dissimilarity_inside_	0.75				0.00	0.03	0.00
							
RII: Species richness_neighboring_	−0.01				0.00	<0.01	0.00
							
Shannon diversity_neighboring_	0.03				0.00	0.00	0.00
							
RII: % Vegetation cover_neighboring_	−0.01				0.00	0.10	0.00
							
Bray–Curtis dissimilarity_neighboring_	0.53				0.00	0.03	0.00
Bray–Curtis dissimilarity_neighboring_	0.48	0.09			0.07	0.11	1.13

## 4 DISCUSSION

### 4.1 Cushion plants

**Figure 7:**
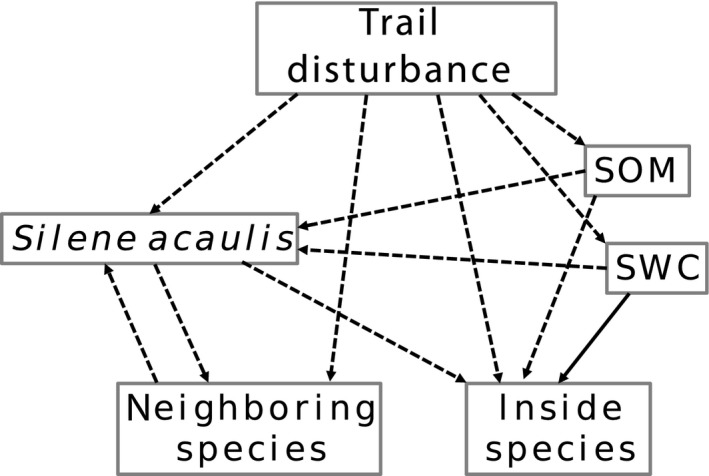
**Conceptual diagram summarizing main findings**. The net (i.e., majority of) effects of trail disturbance, *Silene acaulis* presence*,* soil organic matter (SOM), soil water content (SWC), and neighboring species are indicated (dashed = negative effect; solid = positive effect). Notes on diagram: only individual, not sequential, arrows for each relationship were tested and elevational effects not shown. Notes on parameters: Inside species do not have an effect on *Silene*; the positive effects of disturbance on *Silene* size are not shown because reproduction, density, and small plant size are all negatively affected

We studied systems adjacent to popular hiking trails where trampling is a frequent and relatively high‐intensity disturbance, similar in its severe erosion effects to high‐intensity grazing and landslides. Our data shows that disturbance spurs growth, but reduces population density and reproduction of *Silene* (Figure 7). We suspect that disturbance, either through the mechanical manipulation of cushions or by altering soil conditions, increases adult plant size and reproduction while greatly reducing the ability of smaller plants to survive. This corresponds to the size structure differences we see between disturbed and undisturbed areas, as well as to our findings that population density is lower with disturbance. In the short term, this suggests a positive effect of disturbance on *Silene* growth, however the long‐term effect could be a decline in *Silene* populations as reproduction is decreased and young individuals are unable to survive the impacts of disturbance. The balance between these effects with increased performance of large plants will determine the long‐term net population effects of disturbance, which we cannot judge from our short‐term data. One potential scenario is disturbed populations progressing to larger and larger size structures, with an eventual population decline as these older cushions die off without replacement by younger individuals.

Although other studies have showed that disturbance can negatively affect nurse plant abundance, size, and density (e.g., Ballantyne & Pickering, 2015a,2015b), we are not aware of other studies that have examined responses in cushion plant size structure and reproduction to relatively high disturbance levels. However, past studies also point to changes in abundance and percent cover. Trampling disturbance can reduce the dominant vegetation cover and therefore increase cushion plant cover (Whinam & Chilcott, 2003), as well as cause graminoid species to replace cushion plants growing at lower elevations. Direct trampling on cushions causes portions of *Silene* cushions to die off (Willard & Marr, 1970), which we also observed (*pers. observation*) for cushions growing in the center of the trail. Compared to other alpine tundra species, however, *Silene* cushions can be relatively resistant to trampling (Willard, Cooper, & Forbes, 2007).

### 4.2 Community effects and species interactions

Overall, we found that species diversity and richness within and next to cushions is lower compared to control areas, indicating net competitive interactions between cushions and other plant species (Figure 7). Such negative or neutral interactions have been documented in other studies as well (e.g., de Bello et al., 2011; Dvorsky et al., 2013; Bowman & Swatling‐Holcomb, 2017), but surprised us given that *Silene* has been shown to increase species percent cover and richness (Bonanomi et al., 2015). Although disturbance reduces both species richness and diversity, it has no effect on species interactions, as measured by RII. In undisturbed areas, species richness peaked at middle elevations instead of declining linearly with elevation. We suspect this is due to high levels of biotic competition at low elevations (Supporting Information Table A6 in Appendix S1) and high levels of abiotic stress at upper elevations, as well as an intermediate disturbance effect by grazing at middle elevations. Compared to higher elevations, grazing is most intense at lower elevations and reaches intermediate disturbance levels at middle elevations on mountain slopes, likely increasing species richness in these areas. Furthermore, the unnatural elevational tree line in Switzerland, which has been anthropogenically established due to many centuries of land use and grazing, could cause species richness to be highest at middle elevations where the subalpine–alpine ecotone is reached.

The cushion plant *Silene* has been found to host an increasing number of species at higher elevations (Antonsson, Björk, & Molau, 2009), while also demonstrating greatest facilitative effects on other species at the center of its elevational range (Bonanomi et al., 2015) as well as in abiotically stressful environments (Kjaer, Olsen, & Klanderud, 2017). We therefore expected cushion plants to first, host higher species diversity and richness compared to control areas, and second, maintain this higher diversity in areas where disturbance exerts negative effects. Our careful selection of control areas near to *Silene* cushions that had similar microhabitats is one likely reason that our findings differ from other plant facilitation studies, where control areas are randomly selected near to cushions (e.g., Butterfield et al., 2013). Since cushion plants, including *Silene*, as well as other alpine species, tend to disproportionately occur in favorable microhabitats, we believe that our approach in selecting control areas allows better differentiation of the effects of cushions on other species. This is especially true for alpine environments, which are known to be highly variable in topography, with slight variations in slope and aspect playing a large role in determining species community (Körner, 2003). Completely random choice of control sites can therefore include very different and often less favorable microclimates than those occupied by cushion plants, whereas choosing control areas that match microtopography is likely a more accurate representation of what a species community would look like in the absence of cushion plants. Careful attention to the spatial representation of the microhabitat environment is especially important in ecosystems with cushion plants, as the beneficial microhabitat provided by cushion plants may buffer the effects of climate change (Anthelme, Cavieres, & Dangles, 2014).

Since richness and diversity inside cushions increase with cushion size, we suspect that the positive effects of cushions are only seen once cushions reach a certain size. Comparison of our data with data gathered for another facilitation study (Butterfield et al., 2013) at one of our sites (Val Bercla at Fallerfurgga) shows that our control areas had significantly higher species richness (Supporting Information Figure A8a in Appendix S1), however our data represents the lower end of cushion size distribution (Supporting Information Figure A8b in Appendix S1). As found in many other studies, we would expect a positive correlation between nurse plant size and species richness and diversity (e.g., Incerti et al., 2013; Molenda, Reid, & Lortie, 2012; Tewksbury & Lloyd, 2001; Yang, Chen, Schöb, & Hang, 2017). Smaller nurse plants understandably cannot provide the same microhabitat shelter that larger ones do, and likely act as competitors to other species in the area as they establish. Furthermore, larger plants have had longer time periods in which to accumulate inside species, and their larger surface area increases the chance of establishment by other species. We therefore expected the larger cushions in disturbed environments to have increased richness and diversity, but our results suggest that the overall negative impacts of disturbance on species richness and diversity prevail. In fact, closer examination of richness and diversity as a function of total cushion size shows that disturbed cushions and control areas have a much lower accumulation of species richness and diversity than undisturbed ones (Supporting Information Figure A9 in Appendix S1). Although our model results point to a negative influence of cushion presence on species richness and diversity, disturbance appears to be a stronger driver of these species measures. Disturbance has been found to mediate plant traits that influence facilitative interactions in other systems (Catorci, Malatesta, Velasquez, Tardella, & Zeballos, 2016), however studies examining the impacts of both disturbance and plant traits on facilitative interactions are, to our knowledge, rare. Such relatively high‐intensity disturbances can ultimately prevent plant species from recovering, as shown in a comparable system in the Alaskan arctic tundra (Monz, 2002).

Other studies have shown that facilitative interactions break down at high levels of abiotic stress (for review see Liancourt, Le Bagousse‐Pinguet, Rixen, & Dolezal, 2017; Michalet et al., 2006), implying that positive interactions only increase up to a certain threshold. Considering that trails are sources of frequent disturbances, the lack of facilitative effects in these areas is perhaps not surprising. This is especially true at the species’ upper elevational range limit, where there is increased abiotic stress due to the colder climate. However, we expected to find some indication of facilitation in our off‐trail plots, but competitive interactions dominate here as well. While surprising to us, these results are in agreement with multiple studies that have found lower species richness in cushion plants compared to control areas (e.g., de Bello et al., 2011; Dvorsky et al., 2013), although they contrast with some other alpine facilitation studies (e.g., Butterfield et al., 2013; Callaway et al., 2002).

Our absolute community measures show a negative response to disturbance, but we surprisingly did not detect any significant changes in RII between disturbance types nor along our sampled elevation gradient. Many facilitation studies argue for the use of RII to detect differences in species interactions (e.g., Butterfield et al., 2013; Schöb et al., 2014), however this method does not allow small differences between cushions and control areas to be picked up. Many published facilitation studies observed a much larger difference between cushions and control areas than we did, and therefore the use of RII is reasonable. Using RII to determine if a system is characterized by competitive or facilitative interactions assumes that the relationship between cushion and neighboring communities is proportional, but this relationship undoubtedly changes across climatic regions and ecosystems. The analysis of absolute community measures could therefore present a clearer picture, especially with small differences between cushions and control areas.

Species composition changes have been observed in other disturbed systems (e.g., Monz, 2002; Suding & Goldberg, 2001), and a negative impact of trail disturbance on soils has been found to reduce species richness and abundance (Ballantyne & Pickering, 2015a,2015b; Lucas‐Borja et al., 2011). It is well documented that soil conditions can influence facilitative and competitive species interactions and therefore be drivers of species community composition (e.g., Gross et al., 2009). This holds in our system as well, with SWC increasing species richness and percent vegetation cover. SOM and SWC in turn are both are negatively affected by disturbance and positively affected by the presence of *Silene*. However, the presence of *Silene* cushions does not mitigate this disturbance effect, as seen by decreased species richness and diversity in cushions. These negative impacts of disturbance on the soil environment provide a possible mechanistic explanation of why disturbance reduces species richness and diversity in both cushions and control areas.

Disturbance likely favors plant morphologies that increase resistance to disturbance (e.g., cushion plants with a taproot) and functional groups that can quickly recover after disturbance (e.g., ruderal species). The Swiss Alps have experienced centuries of intermediate disturbance by livestock grazing, resulting in productive and species‐rich meadows above tree line. In fact, reduction in grazing has reduced species richness at these elevations (Dullinger, Dirnböck, Greimler, & Grabherr, 2003). Within these intermediately disturbed areas, we examined areas specifically characterized by relatively high‐intensity disturbance (i.e., hiker trails). We use the terms “undisturbed” and “disturbed” for ease in differentiation of our sampling areas. However, even our “undisturbed” areas experience intermediate levels of disturbance via grazing, while the disturbed areas experience both intermediate grazing and frequent intensity hiker trampling disturbance. Such higher levels of disturbance very likely push these areas above optimal levels of disturbance and into levels of high abiotic stress. Considering that absolute percent vegetation cover in these disturbed areas was still quite high (mean = 48%) compared to undisturbed areas (mean = 58%), it is clear that although our disturbed sites experience a high frequency of human trampling, they are not disturbed enough that they could support only minimal plant life.

With global climate change, species ranges, and therefore biotic interactions, are shifting along latitudinal and elevational gradients. We show that species communities are susceptible to the effects of relatively high‐intensity trampling disturbance, which has negative effects on cushion plants at the population level. In combination with the projected upward expansion of more competitive lower elevation species, this could ultimately lead to sites with high disturbance intensity experiencing rapidly diminishing cushion plant populations at the lower elevational limit. The negative effects of sustained high‐intensity disturbance at upper elevational range limits could ultimately reduce the persistence of upper elevational populations.

## AUTHOR CONTRIBUTIONS

NIC, SW, CR, and DFD designed this study. NIC and AB collected all data, and AB processed all soil samples. NIC conducted all analyses with substantial theoretical and practical suggestions by DFD, SW, and CR. NIC wrote the article with input and revisions by DFD, CR, SW, and AB All authors approved the final version. Our data and R code are freely available at the following Open Science Framework (OSF) site: https://osf.io/6pk3m/. These data are part of the OSF project “Anthropogenic disturbances in alpine ecosystems” (https://doi.org/10.17605/osf.io/gkjv2).
