# Supplementary material for: Local trampling disturbance effects on alpine plant populations and communities: Negative implications for climate change vulnerability
Source: Ecol Evol. 2018 Jul 16;8(16):7921–35. doi: 10.1002/ece3.4276 (PMC6144962; doi:10.1002/ece3.4276)
Supplement: Supplementary file 1 [file ECE3-8-7921-s001.pdf]

APPENDIX S1: Parameters, supplemental results, and supplemental figures

Table A1. Parameters used to test the effects of A) disturbance and elevational level on cushion size and population density and B) disturbance, level, species community indices, and soil conditions on reproduction indicators. The second model set (B) replaces species community indices with soil condition. The full list of models tested is shown in Table B1 in Appendix S2.

A

**Parameter**	**Type**	**Variable**
Disturbance	binary	explanatory
Elevational level	categorical (1:4)	explanatory
SOM	continuous	explanatory
SWC	continuous	explanatory
Silene size	continuous	response
Silene population density	continuous (calculated at plot level)	response

B

**Parameter**	**Type**	**Variable**
Disturbance	binary	explanatory
Elevational level	categorical (1:4)	explanatory
Species Richness	continuous	explanatory
Shannon Diversity Index	continuous	explanatory
% Vegetation Cover	continuous (0:100)	explanatory
Community competitiveness	continuous (1:3)	explanatory
SOM	continuous	explanatory
SWC	continuous	explanatory
Silene fruits per area	continuous	response
Silene relative reproduction	continuous	response

Table A2. Parameters used in models testing the effects of disturbance, elevational level, *Silene acaulis* cushion presence, and sampling area on species community indices. A secondary model set replaces cushion presence with SOM and SWC. The full list of models tested is shown in Table B2 in Appendix S2.

**Parameter**	**Type**	**Variable**
Disturbance	binary	explanatory
Elevational level	categorical (1:4)	explanatory
Silene presence (Cushion)	binary	explanatory
Sampling area	continuous	explanatory
Soil Organic Matter (SOM)	continuous	explanatory
Soil Water Content (SWC)	continuous	explanatory
Species richness	continuous	response
Shannon diversity	continuous	response
Species % cover	continuous	response

Table A3. Parameters used in models testing effects of disturbance and elevation on Soil Organic Matter (SOM) and Soil Water Content (SWC). The full list of models tested is shown in Table B4 in Appendix S2.

**Parameter**	**Type**	**Variable**
Disturbance	binary	explanatory
Elevational level	categorical (1:4)	explanatory
Silene presence (Cushion)	binary	explanatory
Soil Organic Matter	continuous	response
Soil Water Content	continuous	response

Table A4. Parameters used in models testing the effects of disturbance and elevational level on Relative Interaction Indices (RII) and Bray-Curtis Dissimilarity indices. The full list of models tested is shown in Table B5 in Appendix S2.

**Parameter**	**Type**	**Variable**
Disturbance	binary	explanatory
Elevational level	categorical (1:4)	explanatory
RII: species richness	continuous (-1:1)	response
RII: Shannon diversity	continuous (-1:1)	response
RII: species % cover	continuous (-1:1)	response
Bray-Curtis dissimilarity	continuous (0:1)	response

Table A5. Results of most parsimonious models testing the additional effects of Soil Organic Matter (SOM) and Soil Water Content (SWC) on A) *Silene acaulis* cushion parameters and B) inside species community. Light green colors differentiate response variables tested using the same dataset. Interactions (Int(s)) are listed without the corresponding estimates. Elevational level and disturbance are factor variables, with 4 and 2 levels, respectively. Level coefficient values are hence summarized as follows: (+) positive trend, (-) negative trend, or unimodal with a maximum (+) or minimum (-) at levels 2 or 3. All models with Δ AICc values of less than 2 are shown for each response variable with marginal (marg) r^2 and conditional (cond) r^2 listed, and significant p-values (< 0.001***, < 0.01**, < 0.05*) shown above the first listed model within each section. P-values for level indicate that at least one level was significant at < 0.05. The full list of models tested and AICc weights are shown in Tables B1, B2.

A

**Response variable**	**Intercept**	**SOM**	**SWC**	**Disturbance**	**Level**	**Int(s)**	**marg r^2**	**cond r^2**	**Δ AICc**
					*				
Fruits per area	0.06		-0.012	-0.03	unimodal (-)	dist x level x SWC	0.69	0.69	0.00
Relative Reproduction	62.59	-159.39			-	SOM x level	0.27	0.29	0.00
Cushion size	200.92	-31.93	-95.49	-1918.19	-	SOM x SWC x dist x level	0.18	0.20	0.00

B

**Response variable**	**Intercept**	**SOM**	**SWC**	**Disturbance**	**Level**	**Int(s)**	**marg r^2**	**cond r^2**	**Δ AICc**
			*	**					
Species Richness $_{inside}$	5.64	-8.57	6.27	10.60	unimodal (+)	SOM x SWC x dist x level	0.30	0.77	0.00
Shannon Diversity $_{inside}$	1.33						0.00	0.60	0.00
Shannon Diversity $_{inside}$	1.33	0.15					0.05	0.63	1.36
Shannon Diversity $_{inside}$	1.45			-0.25			0.03	0.61	1.59
		*	*		*				
% Vegetation Cover $_{inside}$	26.95	-85.36	75.51	23.03	unimodal (+)	SOM x SWC x dist x level	0.36	0.52	0.00

Table A6. Results of most parsimonious models testing effects of disturbance, elevational level, and *Silene acaulis* presence on community competitiveness within cushion and controls (inside) and within cushion rings and control rings (neighboring). Black bars differentiate different datasets. Interactions (Int(s)) are listed without the corresponding estimates. Elevational level and disturbance are factor variables, with 4 and 2 levels, respectively. Level coefficient values are hence summarized as follows: (+) positive trend, (-) negative trend, or unimodal with a maximum (+) or minimum (-) at levels 2 or 3. All models with Δ AICc values of less than 2 are shown for each response variable with marginal (marg) r^2 and conditional (cond) r^2 listed, and significant p-values (< 0.001***, < 0.01**, < 0.05*) shown above the first listed model within each section. P-values for level indicate that at least one level was significant at < 0.05. The full list of models tested is shown in Table B3 in Appendix S2.

**Response variable**	**Intercept**	**Disturbance**	**Level**	**Cushion**	**Int(s)**	**marg r^2**	**cond r^2**	**Δ AICc**
			*					
Competitiveness $_{inside}$	1.22		-			0.38	0.38	0.00
Competitiveness $_{inside}$	1.18		-	0.08		0.40	0.40	0.34
			*					
Competitiveness $_{neighboring}$	1.21		unimodal (+)			0.52	0.53	0.00
Competitiveness $_{neighboring}$	1.24	-0.06	-			0.54	0.55	

Table A7. Results of most parsimonious models testing effects of disturbance and elevation on Soil Organic Matter (SOM) and Soil Water Content (SWC). Light green colors differentiate response variables tested using the same dataset. Interactions (Int(s)) are listed without the corresponding estimates. Elevational level and disturbance are factor variables, with 4 and 2 levels, respectively. Level coefficient values are hence summarized as follows: (+) positive trend, (-) negative trend, or unimodal with a maximum (+) or minimum (-) at levels 2 or 3. All models with Δ AICc values of less than 2 are shown for each response variable with marginal (marg) r^2 and conditional (cond) r^2 listed, and significant p-values (< 0.001***, < 0.01**, < 0.05*) shown above the first listed model within each section. P-values for level indicate that at least one level was significant at < 0.05. The full list of models tested is shown in Table B4 in Appendix S2.

Response variable	Intercept	Disturbance	Level	Cushion	Int(s)	marg r^2	cond r^2	Δ AICc
			*					
SOM $_{inside}$	25.08	-8.95	unimodal (+)	3.19	dist x level x cush	0.42	0.88	0.00
			*					
SWC $_{inside}$	37.96	-6.85	unimodal (+)	1.35	dist x level x cush	0.39	0.82	0.00

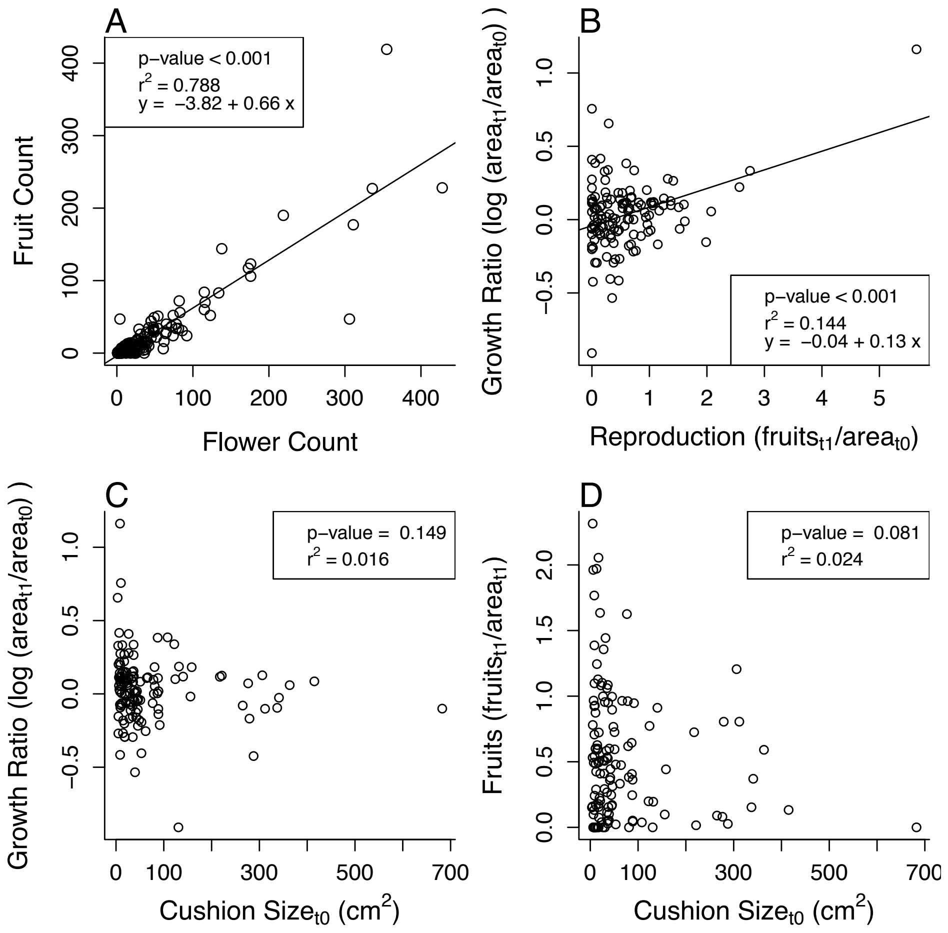

Figure A1. Fruit production and growth rate. Females (A) as well as hermaphrodite (not shown) *Silene acaulis* individuals show a strong and significant correlation between the flowers observed at the beginning of the growing season to the fruits produced at the end of the growing season. Female growth is moderately-well predicted by fruits per area (B), making fruit count a good indicator to use of performance. Both female growth (C) and fruit production (D) have no significant relationship with cushion size of the previous growing season. Data collected (D.F. Doak, W.F. Morris, M.L. Peterson) at Niwot Ridge, Colorado, USA during growing seasons (June – August) in 2015 (t0) and 2016 (t1).

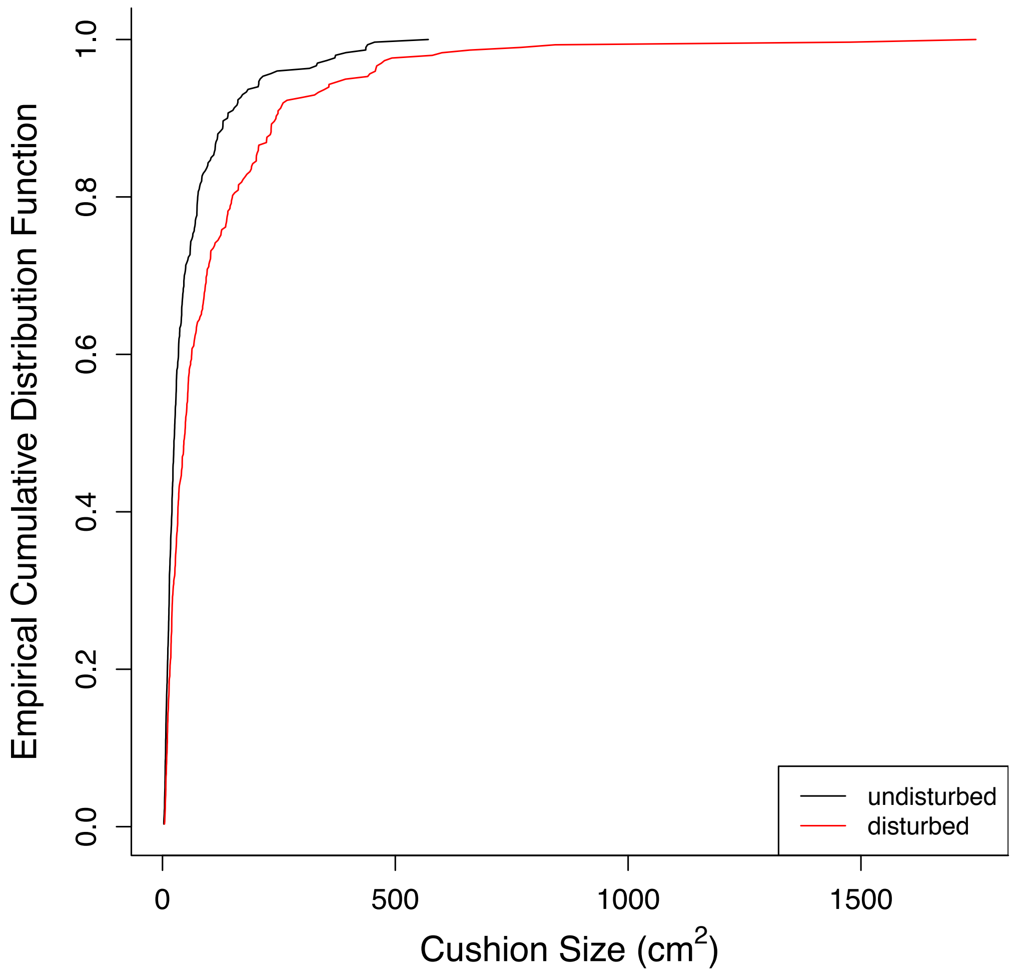

Figure A2. Size distributions. *Silene acaulis* individuals have significantly different size distributions between disturbed and undisturbed areas, as tested with a Two-sample Kolmogorov-Smirnov test (D = 0.21, p-value < 0.001). Disturbed areas have fewer smaller individuals and disturbed cushions reach larger sizes.

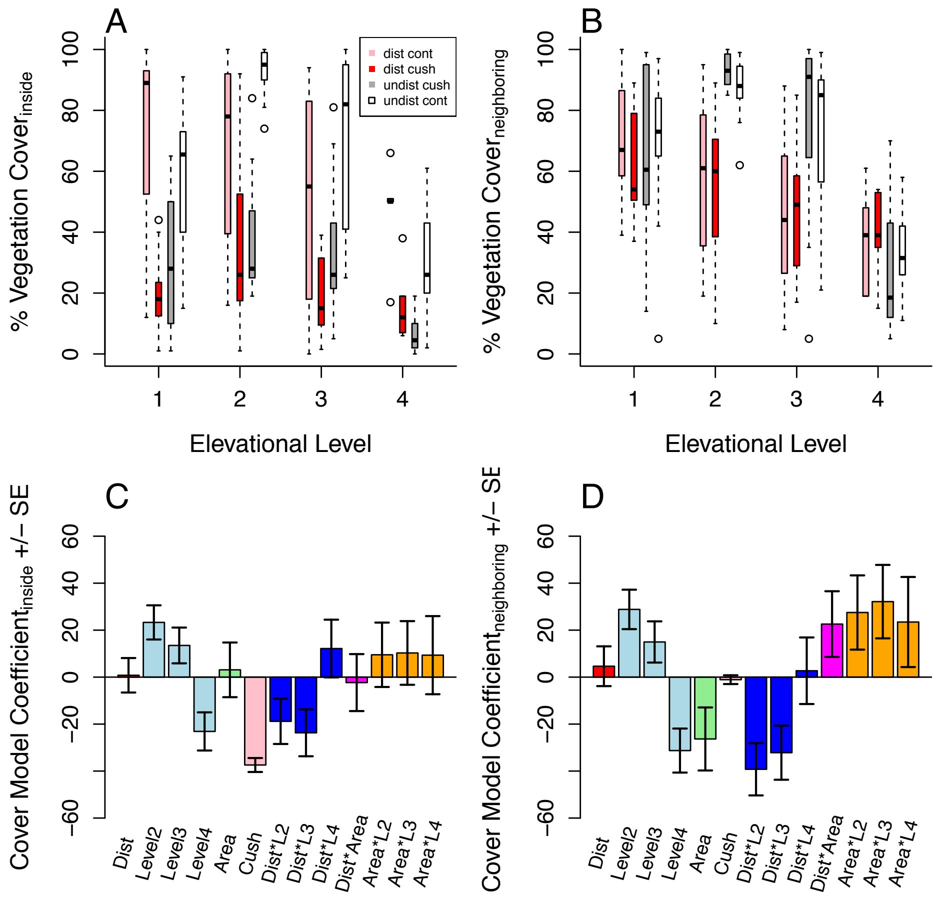

Figure A3. Percent vegetation cover. Disturbance and cushion presence decrease both inside (A) and neighboring (B) species percent vegetation cover (colors for B as in (A)). Legend abbreviations are as follows: dist = disturbed, undist = undisturbed, cush = cushion, cont = control. The best supported model for inside species cover (C) highlights the importance of interactions between disturbance and elevation, which cause decreases in cover at middle elevations (levels 2 and 3), an effect partly mitigated by cushion area. The most parsimonious model for neighboring species cover (D) suggests the same. Both models have an interaction effects between disturbance, elevation, and area, which indicate weaker disturbance effects at low and high elevations and strong effects at middle elevations (see also Table B6 in Appendix S2). Contrasting colors merely differentiate parameters.

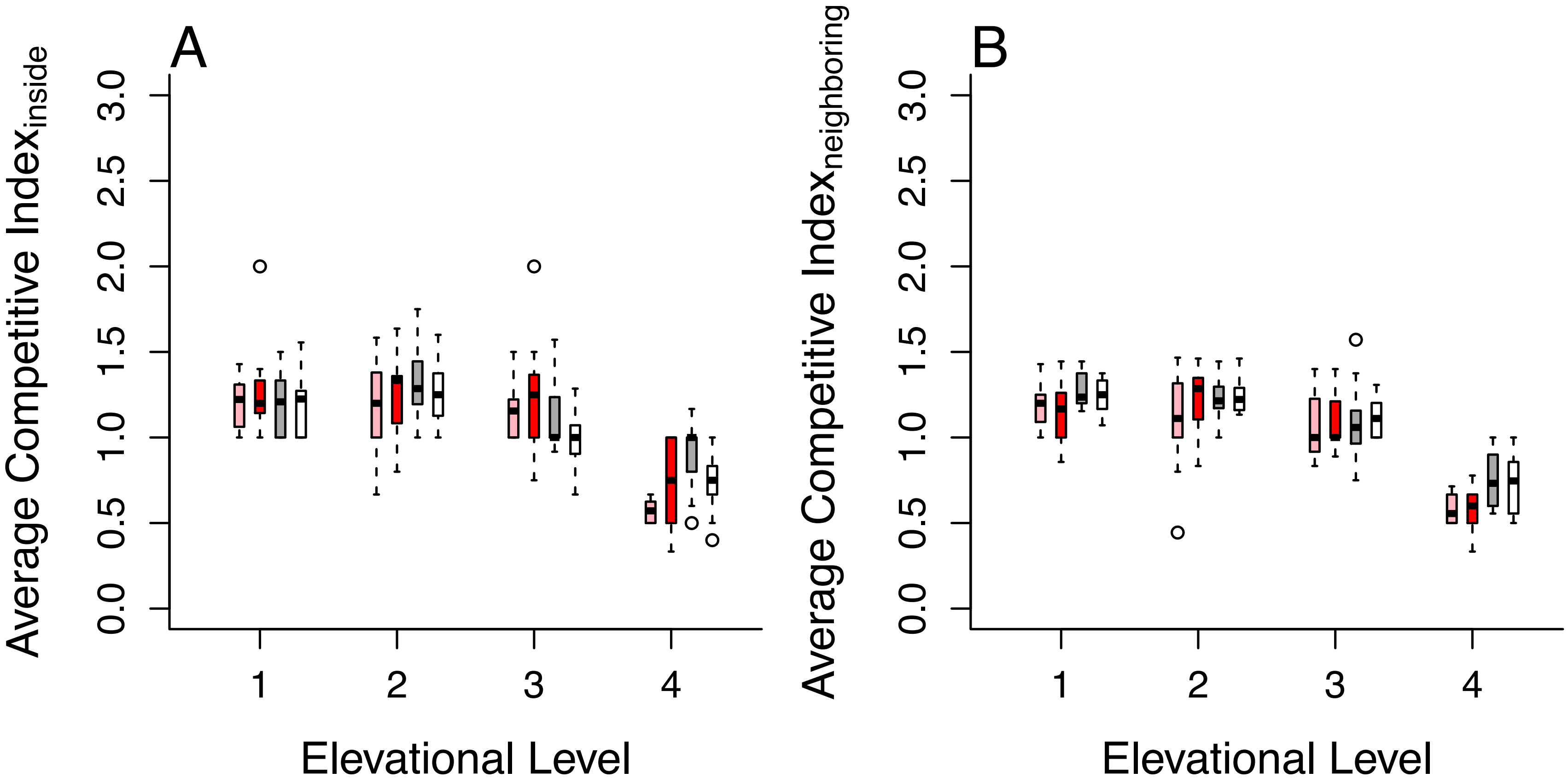

Figure A4. Inside species community competitiveness (A) is highest at low elevations and neighboring community competitiveness (B) is highest at middle elevations. Colors same as in Fig. A3a.

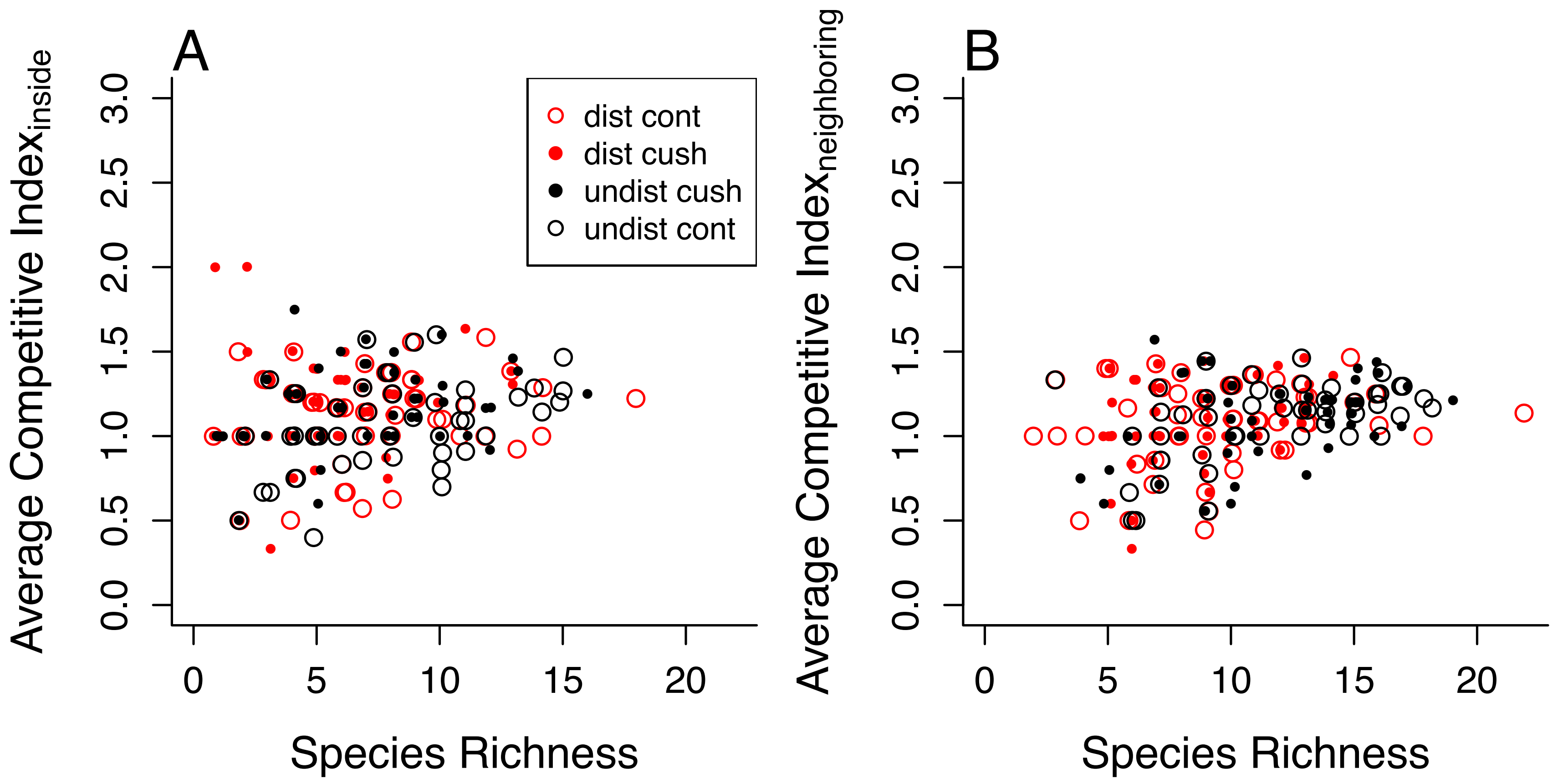

Figure A5. Patterns in community competitiveness are mostly driven by sampling units of median species richness in both inside (A) and neighboring (B) communities, signifying that it is not just highly competitive individual species that drive the observed pattern. Points jittered for clarity. Legend abbreviations are as follows: dist = disturbed, undist = undisturbed, cush = cushion, cont = control.

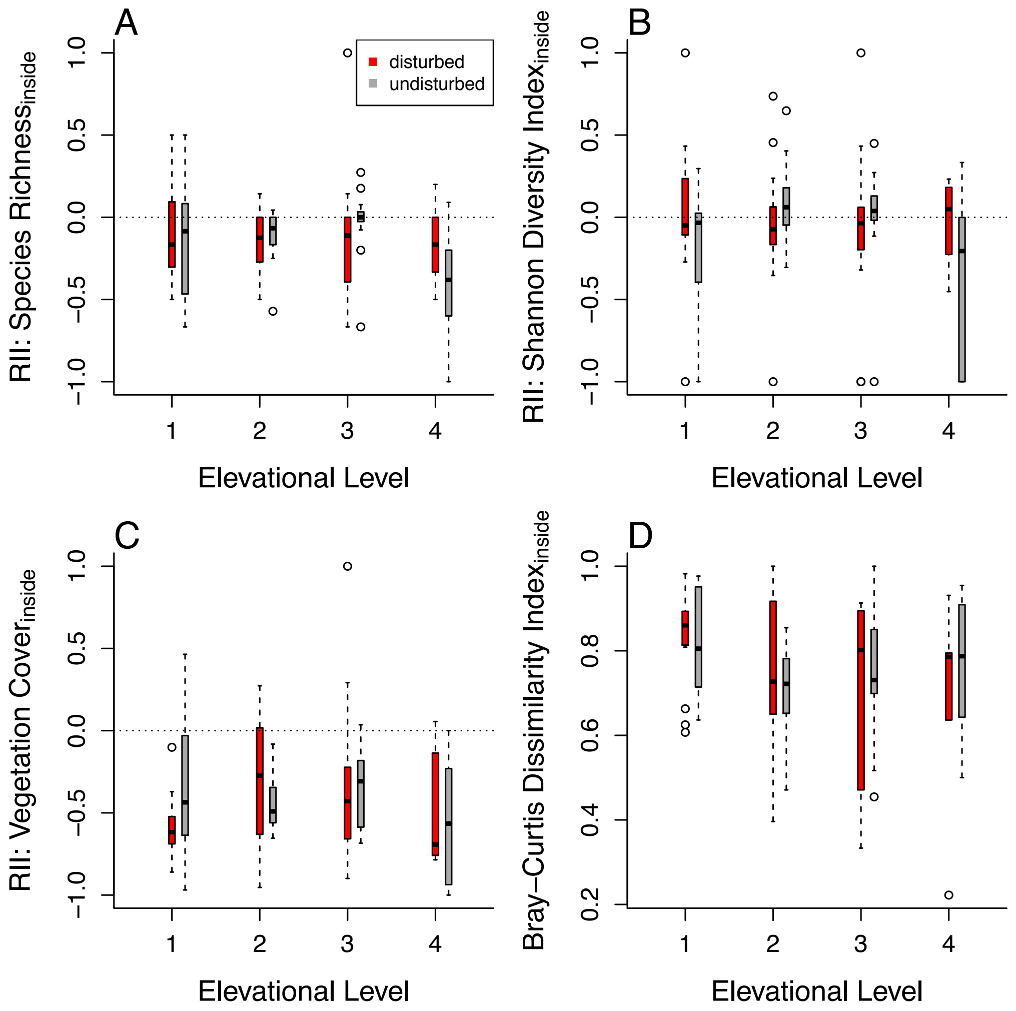

Figure A6. Inside species interactions. The Relative Interaction Index for species richness (A), Shannon diversity (B), percent vegetation cover (C), and the Bray-Curtis Dissimilarity index (D) only include random effects and no effect of disturbance or elevation. Colors all as in (A).

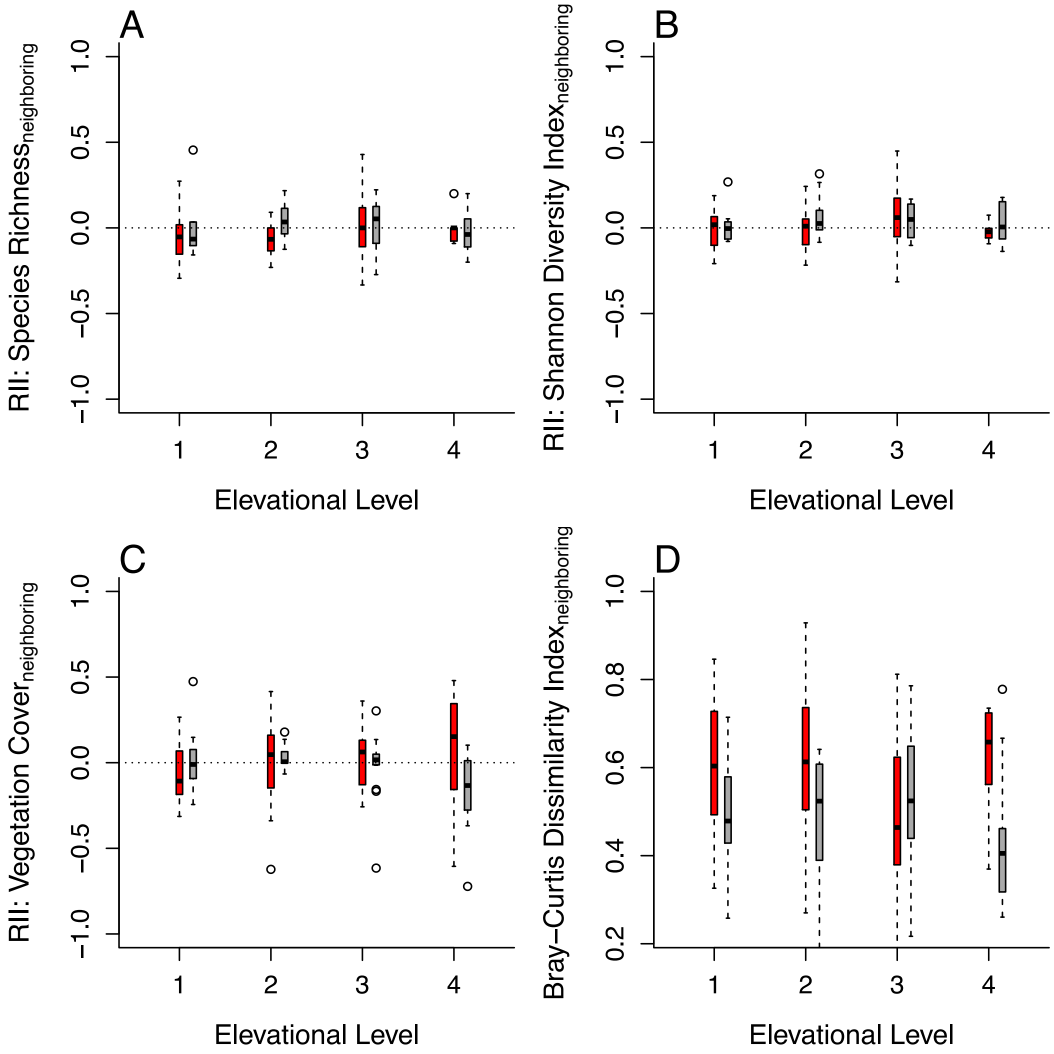

Figure A7. Neighboring species interactions. The Relative Interaction Index for species richness (A), Shannon diversity (B), percent vegetation cover (C), and the Bray-Curtis Dissimilarity index (D) only include random effects and no effect of disturbance or elevation. Colors all as in Fig. A6a.

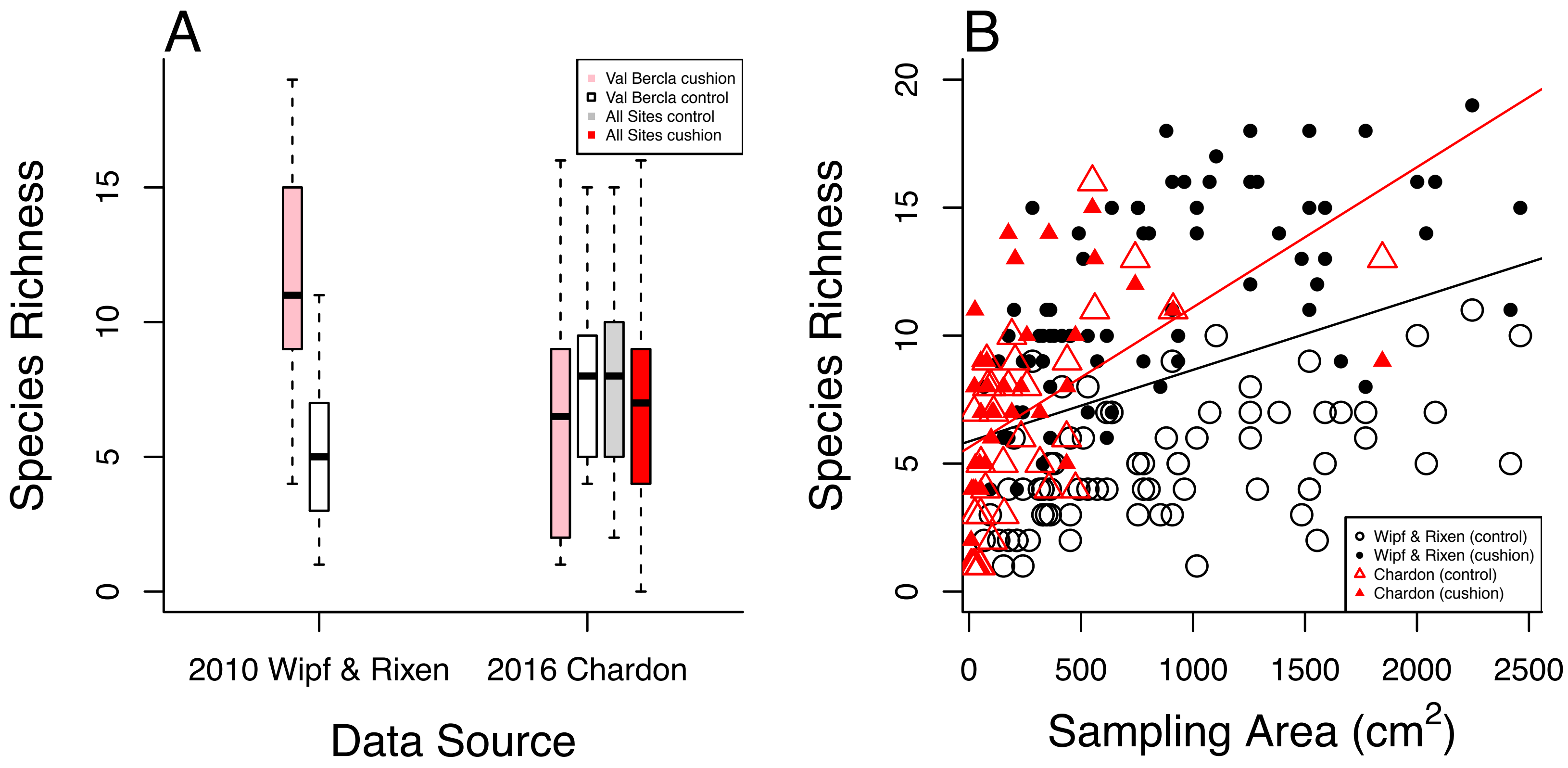

Figure A8. Same-site data comparison. Data comparisons at the same site (Val Bercla at Fallerfurgga) between data we collected in 2016 and Wipf & Rixen collected in 2010 (used in Butterfield et al. 2013) show that (A) we had significantly lower species richness within cushions and significantly higher species richness in our controls than Wipf & Rixen. We suspect that differences in species richness within the controls are attributed to our careful selection of controls that differ from the methods of Wipf & Rixen. The differences in species richness within cushions are likely caused by (B) the discrepancy in the sampled cushion sizes, considering that cushion size increases species richness (see Results). Wipf & Rixen sampled much larger cushions, whereas we sampled proportional to the overall size structure. Linear regression lines including only the fixed effect of sampling area on species richness of both control and cushion shown for both our (p-value < 0.001) and the Wipf & Rixen (p-value < 0.001) data.

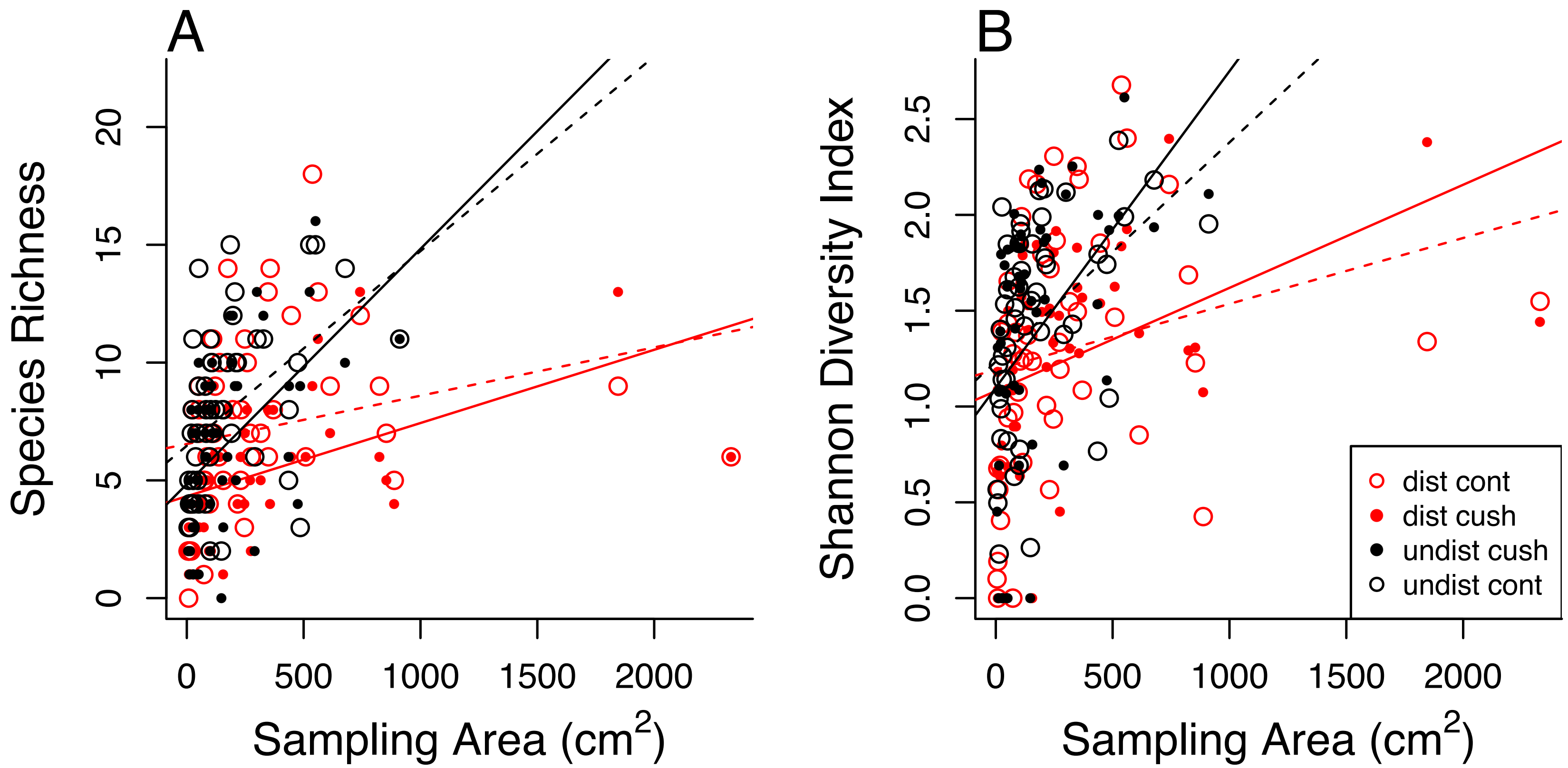

Figure A9. Inside species richness and diversity. Disturbance changes the rate at which inside species richness (A) and diversity (B) accumulate with increasing total size of cushions (i.e. entire area of sampled cushion and not the area taken up by *Silene* itself) and controls. Linear regression lines including only the fixed effect of richness (A) and diversity (B) on cushion size shown and are all significant (p-values < 0.05). Colors in A are as in (B). Legend abbreviations are as follows: dist = disturbed, undist = undisturbed, cush = cushion, cont = control.
