# Supplementary material for: Local trampling disturbance effects on alpine plant populations and communities: Negative implications for climate change vulnerability
Source: Ecol Evol. 2018 Jul 16;8(16):7921–35. doi: 10.1002/ece3.4276 (PMC6144962; doi:10.1002/ece3.4276)
Supplement: Supplementary file 2 [file ECE3-8-7921-s002.pdf]

APPENDIX S2: Full lists of models

Table B1. Full list of Linear Mixed Models to test the effects of disturbance and elevational level on A) cushion size and B) population density. We also tested the effects of C) disturbance, level, and species community indices, as well as D) disturbance, level, and soil conditions on reproduction indicators. Finally, we tested the effects of E) disturbance, level, SOM, and SWC on *Silene* cushion size. Black bars differentiate model sets, and k = number of parameters, n = sample size.

A

modeled parameter	model formula	k	n	AICc	Δ AICc	AICc weight
size	disturbance*level+(1|site)	10	598	7549.91	0.00	1.00
size	disturbance+level+(1|site)	7	598	7582.05	32.14	0.00
size	disturbance+(1|site)	4	598	7598.16	48.25	0.00
size	level+(1|site)	6	598	7605.34	55.43	0.00
size	(1|site)	3	598	7621.59	71.68	0.00

B

modeled parameter	model formula	k	n	AICc	Δ AICc	AICc weight
density	level+(1|site)	6	20	121.40	0.00	0.37
density	disturbance+level+(1|site)	7	20	122.04	0.64	0.27
density	disturbance*level+(1|site)	10	20	122.19	0.79	0.25
density	disturbance+(1|site)	4	20	124.57	3.17	0.08
density	(1|site)	3	20	125.93	4.53	0.04

C

modeled parameter	model formula	k	n	AICc	Δ AICc	AICc weight	species
fruits per area	(1|site)	3	99	223.80	0.00	0.32	inside
fruits per area	competition+(1|site)	4	98	224.48	0.68	0.23	inside
fruits per area	level+(1|site)	6	99	226.61	2.82	0.08	inside
fruits per area	disturbance+(1|site)	4	99	226.79	3.00	0.07	inside
fruits per area	shannon diversity+(1|site)	4	99	227.25	3.45	0.06	inside
fruits per area	disturbance*competition+(1|site)	6	98	227.47	3.67	0.05	inside
fruits per area	level+competition+(1|site)	7	98	227.54	3.74	0.05	inside

fruits per area	disturbance+competition+(1|site)	5	98	227.64	3.84	0.05	inside
fruits per area	level*competition+(1|site)	10	98	229.18	5.39	0.02	inside
fruits per area	species richness+(1|site)	4	99	229.88	6.09	0.02	inside
fruits per area	disturbance+shannon diversity+(1|site)	5	99	229.98	6.18	0.01	inside
fruits per area	disturbance+level+(1|site)	7	99	230.47	6.67	0.01	inside
fruits per area	level+shannon diversity+(1|site)	7	99	231.31	7.52	0.01	inside
fruits per area	disturbance+level+competition+(1|site)	8	98	231.39	7.59	0.01	inside
fruits per area	disturbance+species richness+(1|site)	5	99	232.15	8.35	0.00	inside
fruits per area	level+disturbance*competition+(1|site)	9	98	232.27	8.47	0.00	inside
fruits per area	disturbance*shannon diversity+(1|site)	6	99	232.42	8.62	0.00	inside
fruits per area	vegetation cover+(1|site)	4	99	232.80	9.01	0.00	inside
fruits per area	disturbance*level+(1|site)	10	99	232.88	9.08	0.00	inside
fruits per area	disturbance+level*competition+(1|site)	11	98	233.34	9.55	0.00	inside
fruits per area	disturbance*level+competition+(1|site)	11	98	233.51	9.71	0.00	inside
fruits per area	level+species richness+(1|site)	7	99	234.53	10.73	0.00	inside
fruits per area	disturbance+level+shannon diversity+(1|site)	8	99	235.12	11.33	0.00	inside
fruits per area	disturbance+vegetation cover+(1|site)	5	99	235.16	11.36	0.00	inside
fruits per area	disturbance*species richness+(1|site)	6	99	237.58	13.78	0.00	inside
fruits per area	level+vegetation cover+(1|site)	7	99	237.72	13.92	0.00	inside
fruits per area	disturbance*level+shannon diversity+(1|site)	11	99	237.77	13.98	0.00	inside
fruits per area	disturbance+level+species richness+(1|site)	8	99	238.15	14.35	0.00	inside
fruits per area	level+disturbance*shannon diversity+(1|site)	9	99	238.27	14.48	0.00	inside
fruits per area	disturbance*level*competition+(1|site)	18	98	238.35	14.56	0.00	inside
fruits per area	level*shannon diversity+(1|site)	10	99	239.73	15.94	0.00	inside
fruits per area	disturbance*level+species richness+(1|site)	11	99	241.03	17.24	0.00	inside
fruits per area	disturbance+level+vegetation cover+(1|site)	8	99	241.31	17.52	0.00	inside
fruits per area	disturbance+level*shannon diversity+(1|site)	11	99	243.67	19.87	0.00	inside
fruits per area	disturbance*vegetation cover+(1|site)	6	99	243.76	19.96	0.00	inside
fruits per area	level+disturbance*species richness+(1|site)	9	99	244.33	20.53	0.00	inside
fruits per area	disturbance*level+vegetation cover+(1|site)	11	99	244.42	20.63	0.00	inside

fruits per area	level+disturbance*vegetation cover+(1|site)	9	99	250.98	27.19	0.00	inside
fruits per area	level*species richness+(1|site)	10	99	251.10	27.30	0.00	inside
fruits per area	disturbance+level*species richness+(1|site)	11	99	255.03	31.24	0.00	inside
fruits per area	disturbance*level*shannon diversity+(1|site)	18	99	255.90	32.10	0.00	inside
fruits per area	level*vegetation cover+(1|site)	10	99	260.04	36.25	0.00	inside
fruits per area	disturbance+level*vegetation cover+(1|site)	11	99	264.39	40.60	0.00	inside
fruits per area	disturbance*level*species richness+(1|site)	18	99	280.49	56.69	0.00	inside
fruits per area	disturbance*level*vegetation cover+(1|site)	18	99	300.93	77.14	0.00	inside
relative reproduction	level*competition+(1|site)	10	37	138.83	0.00	0.40	inside
relative reproduction	disturbance+level*competition+(1|site)	11	37	140.32	1.49	0.19	inside
relative reproduction	shannon diversity+(1|site)	4	37	142.80	3.97	0.06	inside
relative reproduction	(1|site)	3	37	143.12	4.29	0.05	inside
relative reproduction	competition+(1|site)	4	37	143.47	4.64	0.04	inside
relative reproduction	disturbance*competition+(1|site)	6	37	143.70	4.87	0.04	inside
relative reproduction	level*shannon diversity+(1|site)	10	37	143.79	4.96	0.03	inside
relative reproduction	disturbance+(1|site)	4	37	144.19	5.36	0.03	inside
relative reproduction	disturbance+shannon diversity+(1|site)	5	37	144.70	5.87	0.02	inside
relative reproduction	disturbance+competition+(1|site)	5	37	144.74	5.91	0.02	inside
relative reproduction	level+shannon diversity+(1|site)	7	37	145.02	6.19	0.02	inside
relative reproduction	level+(1|site)	6	37	145.14	6.31	0.02	inside
relative reproduction	disturbance*shannon diversity+(1|site)	6	37	145.56	6.73	0.01	inside
relative reproduction	disturbance+level+(1|site)	7	37	145.69	6.86	0.01	inside
relative reproduction	disturbance+level*shannon diversity+(1|site)	11	37	145.87	7.04	0.01	inside
relative reproduction	level+competition+(1|site)	7	37	146.12	7.29	0.01	inside
relative reproduction	level+disturbance*competition+(1|site)	9	37	146.58	7.75	0.01	inside
relative reproduction	disturbance+level+shannon diversity+(1|site)	8	37	146.88	8.05	0.01	inside
relative reproduction	disturbance+level+competition+(1|site)	8	37	146.95	8.12	0.01	inside
relative reproduction	species richness+(1|site)	4	37	147.29	8.46	0.01	inside
relative reproduction	level+disturbance*shannon diversity+(1|site)	9	37	148.14	9.31	0.00	inside

relative reproduction	disturbance+species richness+(1|site)	5	37	149.04	10.21	0.00	inside
relative reproduction	level+species richness+(1|site)	7	37	149.86	11.03	0.00	inside
relative reproduction	vegetation cover+(1|site)	4	37	149.98	11.15	0.00	inside
relative reproduction	disturbance+level+species richness+(1|site)	8	37	151.32	12.49	0.00	inside
relative reproduction	disturbance+vegetation cover+(1|site)	5	37	151.81	12.98	0.00	inside
relative reproduction	disturbance*species richness+(1|site)	6	37	152.94	14.11	0.00	inside
relative reproduction	level+vegetation cover+(1|site)	7	37	153.31	14.48	0.00	inside
relative reproduction	disturbance+level+vegetation cover+(1|site)	8	37	154.83	16.00	0.00	inside
relative reproduction	level+disturbance*species richness+(1|site)	9	37	154.89	16.06	0.00	inside
relative reproduction	level*species richness+(1|site)	10	37	159.36	20.53	0.00	inside
relative reproduction	disturbance*vegetation cover+(1|site)	6	37	159.74	20.91	0.00	inside
relative reproduction	disturbance+level*species richness+(1|site)	11	37	161.23	22.40	0.00	inside
relative reproduction	level+disturbance*vegetation cover+(1|site)	9	37	162.80	23.97	0.00	inside
relative reproduction	level*vegetation cover+(1|site)	10	37	171.77	32.94	0.00	inside
relative reproduction	disturbance+level*vegetation cover+(1|site)	11	37	174.21	35.38	0.00	inside
fruits per area	disturbance*shannon diversity+(1|site)	6	99	218.30	0.00	0.66	neighboring
fruits per area	shannon diversity+(1|site)	4	99	223.64	5.34	0.05	neighboring
fruits per area	competition+(1|site)	4	99	223.71	5.41	0.04	neighboring
fruits per area	(1|site)	3	99	223.80	5.49	0.04	neighboring
fruits per area	disturbance+shannon diversity+(1|site)	5	99	223.80	5.50	0.04	neighboring
fruits per area	level*shannon diversity+(1|site)	10	99	223.94	5.63	0.04	neighboring
fruits per area	disturbance*level*shannon diversity+(1|site)	18	99	224.08	5.77	0.04	neighboring
fruits per area	disturbance*competition+(1|site)	6	99	225.41	7.10	0.02	neighboring
fruits per area	level+(1|site)	6	99	226.61	8.31	0.01	neighboring
fruits per area	disturbance+competition+(1|site)	5	99	226.71	8.41	0.01	neighboring
fruits per area	disturbance+(1|site)	4	99	226.79	8.49	0.01	neighboring
fruits per area	disturbance+level*shannon diversity+(1|site)	11	99	226.96	8.65	0.01	neighboring
fruits per area	disturbance+species richness+(1|site)	5	99	228.08	9.77	0.00	neighboring
fruits per area	level*competition+(1|site)	10	99	228.17	9.86	0.00	neighboring

fruits per area	species richness+(1|site)	4	99	228.19	9.89	0.00	neighboring
fruits per area	level+competition+(1|site)	7	99	228.47	10.16	0.00	neighboring
fruits per area	level+disturbance*shannon diversity+(1|site)	9	99	228.56	10.26	0.00	neighboring
fruits per area	level+shannon diversity+(1|site)	7	99	229.04	10.74	0.00	neighboring
fruits per area	disturbance+level+(1|site)	7	99	230.47	12.16	0.00	neighboring
fruits per area	disturbance+level+shannon diversity+(1|site)	8	99	231.30	12.99	0.00	neighboring
fruits per area	disturbance*species richness+(1|site)	6	99	231.48	13.17	0.00	neighboring
fruits per area	vegetation cover+(1|site)	4	99	231.81	13.50	0.00	neighboring
fruits per area	level+disturbance*competition+(1|site)	9	99	232.12	13.81	0.00	neighboring
fruits per area	disturbance+level*competition+(1|site)	11	99	232.40	14.10	0.00	neighboring
fruits per area	disturbance+level+competition+(1|site)	8	99	232.46	14.15	0.00	neighboring
fruits per area	disturbance+vegetation cover+(1|site)	5	99	232.62	14.31	0.00	neighboring
fruits per area	disturbance*level+(1|site)	10	99	232.88	14.57	0.00	neighboring
fruits per area	level+species richness+(1|site)	7	99	233.77	15.46	0.00	neighboring
fruits per area	disturbance*level*competition+(1|site)	18	99	234.74	16.43	0.00	neighboring
fruits per area	disturbance*level+shannon diversity+(1|site)	11	99	234.81	16.51	0.00	neighboring
fruits per area	disturbance*level+competition+(1|site)	11	99	235.20	16.89	0.00	neighboring
fruits per area	disturbance+level+species richness+(1|site)	8	99	235.97	17.67	0.00	neighboring
fruits per area	level+vegetation cover+(1|site)	7	99	238.03	19.73	0.00	neighboring
fruits per area	disturbance*level+species richness+(1|site)	11	99	239.54	21.24	0.00	neighboring
fruits per area	level+disturbance*species richness+(1|site)	9	99	240.27	21.96	0.00	neighboring
fruits per area	level*species richness+(1|site)	10	99	240.52	22.22	0.00	neighboring
fruits per area	disturbance+level+vegetation cover+(1|site)	8	99	240.85	22.54	0.00	neighboring
fruits per area	disturbance*vegetation cover+(1|site)	6	99	241.48	23.17	0.00	neighboring
fruits per area	disturbance+level*species richness+(1|site)	11	99	243.65	25.34	0.00	neighboring
fruits per area	disturbance*level+vegetation cover+(1|site)	11	99	244.49	26.18	0.00	neighboring
fruits per area	level+disturbance*vegetation cover+(1|site)	9	99	250.41	32.10	0.00	neighboring
fruits per area	disturbance*level*species richness+(1|site)	18	99	261.26	42.95	0.00	neighboring
fruits per area	level*vegetation cover+(1|site)	10	99	265.55	47.25	0.00	neighboring
fruits per area	disturbance+level*vegetation cover+(1|site)	11	99	269.26	50.95	0.00	neighboring

fruits per area	disturbance*level*vegetation cover+(1|site)	18	99	309.02	90.71	0.00	neighboring
relative reproduction	level*competition+(1|site)	10	37	127.60	0.00	0.73	neighboring
relative reproduction	disturbance+level*competition+(1|site)	11	37	129.61	2.01	0.27	neighboring
relative reproduction	level*shannon diversity+(1|site)	10	37	139.61	12.01	0.00	neighboring
relative reproduction	competition+(1|site)	4	37	140.64	13.04	0.00	neighboring
relative reproduction	disturbance*competition+(1|site)	6	37	141.05	13.45	0.00	neighboring
relative reproduction	disturbance+level*shannon diversity+(1|site)	11	37	141.54	13.94	0.00	neighboring
relative reproduction	disturbance+competition+(1|site)	5	37	141.90	14.29	0.00	neighboring
relative reproduction	(1|site)	3	37	143.12	15.52	0.00	neighboring
relative reproduction	level+competition+(1|site)	7	37	143.27	15.67	0.00	neighboring
relative reproduction	level+disturbance*competition+(1|site)	9	37	143.80	16.20	0.00	neighboring
relative reproduction	shannon diversity+(1|site)	4	37	144.05	16.45	0.00	neighboring
relative reproduction	disturbance+(1|site)	4	37	144.19	16.58	0.00	neighboring
relative reproduction	disturbance+level+competition+(1|site)	8	37	144.65	17.05	0.00	neighboring
relative reproduction	level+(1|site)	6	37	145.14	17.54	0.00	neighboring
relative reproduction	disturbance*shannon diversity+(1|site)	6	37	145.51	17.90	0.00	neighboring
relative reproduction	disturbance+shannon diversity+(1|site)	5	37	145.55	17.95	0.00	neighboring
relative reproduction	vegetation cover+(1|site)	4	37	145.58	17.98	0.00	neighboring
relative reproduction	disturbance+level+(1|site)	7	37	145.69	18.09	0.00	neighboring
relative reproduction	species richness+(1|site)	4	37	146.79	19.19	0.00	neighboring
relative reproduction	level+shannon diversity+(1|site)	7	37	146.85	19.25	0.00	neighboring
relative reproduction	disturbance+level+shannon diversity+(1|site)	8	37	147.47	19.87	0.00	neighboring
relative reproduction	level+disturbance*shannon diversity+(1|site)	9	37	147.61	20.01	0.00	neighboring
relative reproduction	disturbance+vegetation cover+(1|site)	5	37	147.76	20.16	0.00	neighboring
relative reproduction	disturbance+species richness+(1|site)	5	37	148.63	21.03	0.00	neighboring
relative reproduction	level+vegetation cover+(1|site)	7	37	148.85	21.25	0.00	neighboring
relative reproduction	level+species richness+(1|site)	7	37	149.98	22.38	0.00	neighboring
relative reproduction	disturbance+level+vegetation cover+(1|site)	8	37	151.36	23.75	0.00	neighboring
relative reproduction	disturbance+level+species richness+(1|site)	8	37	151.60	24.00	0.00	neighboring

relative reproduction	disturbance*species richness+(1|site)	6	37	152.50	24.90	0.00	neighboring
relative reproduction	disturbance*vegetation cover+(1|site)	6	37	154.69	27.09	0.00	neighboring
relative reproduction	level+disturbance*species richness+(1|site)	9	37	155.61	28.01	0.00	neighboring
relative reproduction	level*species richness+(1|site)	10	37	158.44	30.84	0.00	neighboring
relative reproduction	level+disturbance*vegetation cover+(1|site)	9	37	158.49	30.88	0.00	neighboring
relative reproduction	disturbance+level*species richness+(1|site)	11	37	161.32	33.72	0.00	neighboring
relative reproduction	level*vegetation cover+(1|site)	10	37	166.90	39.30	0.00	neighboring
relative reproduction	disturbance+level*vegetation cover+(1|site)	11	37	169.05	41.45	0.00	neighboring

D

**modeled formula**	**model formula**	**k**	**n**	**AICc**	**Δ AICc**	**AICc weight**
fruits per area	disturbance*level*SWC+(1|site)	18	57	144.05	0.00	0.93
fruits per area	disturbance*level*SWC+SOM+(1|site)	19	57	149.12	5.08	0.07
fruits per area	(1|site)	3	57	162.66	18.61	0.00
fruits per area	level+(1|site)	6	57	163.78	19.73	0.00
fruits per area	disturbance+(1|site)	4	57	164.97	20.92	0.00
fruits per area	disturbance+SOM+(1|site)	5	57	165.82	21.77	0.00
fruits per area	SOM+(1|site)	4	57	165.93	21.89	0.00
fruits per area	SWC+(1|site)	4	57	166.76	22.72	0.00
fruits per area	disturbance+level+(1|site)	7	57	166.93	22.88	0.00
fruits per area	disturbance+SWC+(1|site)	5	57	168.18	24.13	0.00
fruits per area	disturbance*level+(1|site)	10	57	168.22	24.17	0.00
fruits per area	level+SOM+(1|site)	7	57	168.31	24.27	0.00
fruits per area	disturbance*SOM+(1|site)	6	57	168.48	24.43	0.00
fruits per area	disturbance+SOM+SWC+(1|site)	6	57	168.60	24.56	0.00
fruits per area	level+SWC+(1|site)	7	57	168.71	24.66	0.00
fruits per area	SOM+SWC+(1|site)	5	57	168.89	24.85	0.00
fruits per area	disturbance+level+SOM+(1|site)	8	57	170.47	26.42	0.00
fruits per area	disturbance*SWC+(1|site)	6	57	171.05	27.00	0.00

fruits per area	disturbance*SOM+SWC+(1|site)	7	57	171.39	27.35	0.00
fruits per area	disturbance*SOM+SWC*(1|site)	7	57	171.39	27.35	0.00
fruits per area	level+SOM+SWC+(1|site)	8	57	171.49	27.45	0.00
fruits per area	disturbance+level+SWC+(1|site)	8	57	171.58	27.53	0.00
fruits per area	SOM*SWC+(1|site)	6	57	172.15	28.11	0.00
fruits per area	disturbance*level+SWC+(1|site)	11	57	172.21	28.16	0.00
fruits per area	disturbance*level+SOM+(1|site)	11	57	172.86	28.82	0.00
fruits per area	level+disturbance*SOM+(1|site)	9	57	173.14	29.10	0.00
fruits per area	disturbance+level+SOM+SWC+(1|site)	9	57	173.33	29.29	0.00
fruits per area	level+disturbance*SWC+SOM+(1|site)	10	57	173.93	29.89	0.00
fruits per area	level+disturbance*SWC+(1|site)	9	57	173.94	29.90	0.00
fruits per area	disturbance*level+SOM+SWC+(1|site)	12	57	174.36	30.32	0.00
fruits per area	level*SOM+(1|site)	10	57	175.06	31.01	0.00
fruits per area	level+disturbance*SOM+SWC+(1|site)	10	57	175.41	31.36	0.00
fruits per area	disturbance*level*SOM+(1|site)	18	57	176.70	32.65	0.00
fruits per area	disturbance+level+SOM*SWC+(1|site)	10	57	176.91	32.86	0.00
fruits per area	level+disturbance+SOM*SWC+(1|site)	10	57	176.91	32.86	0.00
fruits per area	level*SOM+SWC+(1|site)	11	57	177.12	33.08	0.00
fruits per area	level*SOM+SWC+(1|site)	11	57	177.12	33.08	0.00
fruits per area	disturbance*level*SOM+SWC+(1|site)	19	57	177.72	33.68	0.00
fruits per area	disturbance+level*SOM+(1|site)	11	57	178.20	34.15	0.00
fruits per area	level*SWC+(1|site)	10	57	178.44	34.39	0.00
fruits per area	disturbance*level+SOM*SWC+(1|site)	13	57	178.72	34.67	0.00
fruits per area	disturbance+level*SOM+SWC+(1|site)	12	57	180.25	36.20	0.00
fruits per area	disturbance+level*SWC+(1|site)	11	57	181.16	37.11	0.00
fruits per area	disturbance+level*SWC+SOM+(1|site)	12	57	182.59	38.55	0.00
fruits per area	level*SOM*SWC+(1|site)	18	57	184.51	40.47	0.00
relative reproduction	disturbance*SOM+(1|site)	6	17	62.74	0.00	0.37
relative reproduction	SWC+(1|site)	4	17	65.62	2.88	0.09

relative reproduction	SOM*SWC+(1|site)	6	17	65.83	3.10	0.08
relative reproduction	SOM+(1|site)	4	17	65.97	3.23	0.07
relative reproduction	disturbance*SOM+SWC+(1|site)	7	17	66.29	3.56	0.06
relative reproduction	disturbance*SOM+SWC*(1|site)	7	17	66.29	3.56	0.06
relative reproduction	level*SOM+(1|site)	8	17	66.79	4.05	0.05
relative reproduction	disturbance+SOM+(1|site)	5	17	67.00	4.27	0.04
relative reproduction	disturbance+(1|site)	4	17	67.64	4.90	0.03
relative reproduction	disturbance+SWC+(1|site)	5	17	67.69	4.96	0.03
relative reproduction	SOM+SWC+(1|site)	5	17	68.08	5.35	0.03
relative reproduction	(1|site)	3	17	68.34	5.60	0.02
relative reproduction	disturbance*SWC+(1|site)	6	17	68.98	6.24	0.02
relative reproduction	disturbance+SOM+SWC+(1|site)	6	17	69.86	7.12	0.01
relative reproduction	level+SWC+(1|site)	6	17	70.30	7.57	0.01
relative reproduction	level+SOM+(1|site)	6	17	71.23	8.49	0.01
relative reproduction	disturbance+level+(1|site)	6	17	71.47	8.73	0.00
relative reproduction	level+(1|site)	5	17	72.29	9.56	0.00
relative reproduction	level+disturbance*SOM+(1|site)	8	17	72.70	9.97	0.00
relative reproduction	disturbance+level*SOM+(1|site)	9	17	73.30	10.57	0.00
relative reproduction	level*SOM+SWC+(1|site)	9	17	73.62	10.88	0.00
relative reproduction	level*SOM+SWC+(1|site)	9	17	73.62	10.88	0.00
relative reproduction	disturbance+level+SWC+(1|site)	7	17	74.17	11.43	0.00
relative reproduction	disturbance+level+SOM+(1|site)	7	17	74.26	11.53	0.00
relative reproduction	level+SOM+SWC+(1|site)	7	17	74.61	11.87	0.00
relative reproduction	level*SWC+(1|site)	8	17	75.95	13.21	0.00
relative reproduction	level+disturbance*SWC+(1|site)	8	17	78.04	15.30	0.00
relative reproduction	disturbance+level+SOM+SWC+(1|site)	8	17	79.17	16.43	0.00
relative reproduction	disturbance+level+SOM*SWC+(1|site)	9	17	80.11	17.37	0.00
relative reproduction	level+disturbance+SOM*SWC+(1|site)	9	17	80.11	17.37	0.00
relative reproduction	level+disturbance*SOM+SWC+(1|site)	9	17	80.11	17.37	0.00
relative reproduction	disturbance+level*SWC+(1|site)	9	17	83.24	20.51	0.00

relative reproduction	disturbance+level*SOM+SWC+(1|site)	10	17	83.41	20.68	0.00
relative reproduction	level+disturbance*SWC+SOM+(1|site)	9	17	83.46	20.72	0.00
relative reproduction	disturbance+level*SWC+SOM+(1|site)	10	17	93.47	30.73	0.00

E

modeled parameter	model formula	k	n	AICc	∆ AICc	AICc weight
size	disturbance*level*SOM+SWC+(1|site)	19	57	654.80	0.00	0.89
size	disturbance*level*SWC+SOM+(1|site)	19	57	659.55	4.74	0.08
size	disturbance*level*SOM+(1|site)	18	57	662.47	7.67	0.02
size	disturbance*level*SWC+(1|site)	18	57	667.59	12.79	0.00
size	level*SOM*SWC+(1|site)	18	57	668.89	14.09	0.00
size	disturbance*level+SOM*SWC+(1|site)	13	57	720.29	65.48	0.00
size	disturbance*level+SOM+SWC+(1|site)	12	57	727.66	72.85	0.00
size	disturbance+level*SWC+SOM+(1|site)	12	57	730.38	75.58	0.00
size	disturbance+level*SOM+SWC+(1|site)	12	57	730.91	76.11	0.00
size	disturbance*level+SWC+(1|site)	11	57	736.36	81.55	0.00
size	disturbance*level+SOM+(1|site)	11	57	737.81	83.01	0.00
size	disturbance+level*SWC+(1|site)	11	57	738.90	84.10	0.00
size	level*SOM+SWC+(1|site)	11	57	740.02	85.21	0.00
size	level*SOM+SWC+(1|site)	11	57	740.02	85.21	0.00
size	disturbance+level*SOM+(1|site)	11	57	740.87	86.07	0.00
size	disturbance*level+(1|site)	10	57	745.21	90.41	0.00
size	level*SWC+(1|site)	10	57	748.10	93.29	0.00
size	level+disturbance*SOM+SWC+(1|site)	10	57	749.49	94.69	0.00
size	level+disturbance*SWC+SOM+(1|site)	10	57	749.64	94.84	0.00
size	level*SOM+(1|site)	10	57	749.91	95.10	0.00
size	disturbance+level+SOM*SWC+(1|site)	10	57	750.61	95.80	0.00
size	level+disturbance+SOM*SWC+(1|site)	10	57	750.61	95.80	0.00
size	disturbance+level+SOM+SWC+(1|site)	9	57	758.35	103.55	0.00
size	level+disturbance*SWC+(1|site)	9	57	758.96	104.15	0.00

size	level+disturbance*SOM+(1|site)	9	57	760.06	105.26	0.00
size	disturbance+level+SWC+(1|site)	8	57	768.28	113.48	0.00
size	disturbance+level+SOM+(1|site)	8	57	769.68	114.88	0.00
size	level+SOM+SWC+(1|site)	8	57	769.70	114.90	0.00
size	disturbance*SOM+SWC+(1|site)	7	57	776.34	121.54	0.00
size	disturbance*SOM+SWC*(1|site)	7	57	776.34	121.54	0.00
size	disturbance+level+(1|site)	7	57	777.15	122.34	0.00
size	level+SWC+(1|site)	7	57	778.21	123.41	0.00
size	level+SOM+(1|site)	7	57	779.97	125.17	0.00
size	disturbance*SWC+(1|site)	6	57	785.34	130.53	0.00
size	disturbance+SOM+SWC+(1|site)	6	57	785.62	130.82	0.00
size	disturbance*SOM+(1|site)	6	57	786.40	131.59	0.00
size	SOM*SWC+(1|site)	6	57	789.11	134.31	0.00
size	level+(1|site)	6	57	789.46	134.66	0.00
size	disturbance+SWC+(1|site)	5	57	795.33	140.52	0.00
size	disturbance+SOM+(1|site)	5	57	796.63	141.83	0.00
size	SOM+SWC+(1|site)	5	57	797.56	142.75	0.00
size	disturbance+(1|site)	4	57	804.29	149.48	0.00
size	SWC+(1|site)	4	57	806.28	151.48	0.00
size	SOM+(1|site)	4	57	807.74	152.94	0.00
size	(1|site)	3	57	817.31	162.51	0.00

Table B2. Full list of Linear Mixed Models to test effects of A) disturbance, elevational level, *Silene acaulis* cushion presence, and sampling area on species community indices. B) A secondary model set replaces cushion presence with SOM and SWC, and could only be tested on inside species. Black bars differentiate model sets, and k = number of parameters, n = sample size.

A

modeled parameter	model formula	k	n	AICc	Δ AICc	AICc weight	species
species richness	disturbance*level*area+cushion+(1|site/cushion ID)	20	198	947.01	0.00	1.00	inside
species richness	disturbance+level*area+cushion+(1|site/cushion ID)	13	198	960.89	13.88	0.00	inside
species richness	disturbance*level+area+cushion+(1|site/cushion ID)	13	198	966.23	19.22	0.00	inside
species richness	level+disturbance*area+cushion+(1|site/cushion ID)	11	198	967.42	20.41	0.00	inside
species richness	cushion+area*level+(1|site/cushion ID)	12	198	970.79	23.78	0.00	inside
species richness	disturbance+level+area+cushion+(1|site/cushion ID)	10	198	974.30	27.29	0.00	inside
species richness	cushion+area+level+(1|site/cushion ID)	9	198	983.60	36.59	0.00	inside
species richness	cushion*level+area+(1|site/cushion ID)	12	198	983.98	36.97	0.00	inside
species richness	disturbance*area+cushion+(1|site/cushion ID)	8	198	990.02	43.01	0.00	inside
species richness	disturbance*level*cushion+(1|site/cushion ID)	19	200	992.19	45.18	0.00	inside
species richness	disturbance*level+cushion+(1|site/cushion ID)	12	200	995.16	48.15	0.00	inside
species richness	disturbance+cushion+area+(1|site/cushion ID)	7	198	996.95	49.94	0.00	inside
species richness	disturbance*cushion+area+(1|site/cushion ID)	8	198	997.45	50.44	0.00	inside
species richness	disturbance+level+cushion+(1|site/cushion ID)	9	200	1001.00	53.99	0.00	inside
species richness	disturbance+level*cushion+(1|site/cushion ID)	12	200	1001.41	54.41	0.00	inside
species richness	cushion+area+(1|site/cushion ID)	6	198	1001.79	54.78	0.00	inside
species richness	level+disturbance*cushion+(1|site/cushion ID)	10	200	1001.80	54.79	0.00	inside
species richness	cushion+level+(1|site/cushion ID)	8	200	1004.37	57.36	0.00	inside
species richness	cushion*level+(1|site/cushion ID)	11	200	1004.71	57.70	0.00	inside
species richness	disturbance*level+(1|site/cushion ID)	11	200	1018.92	71.91	0.00	inside
species richness	disturbance+cushion+(1|site/cushion ID)	6	200	1020.31	73.30	0.00	inside
species richness	disturbance*cushion+(1|site/cushion ID)	7	200	1021.04	74.03	0.00	inside
species richness	cushion+(1|site/cushion ID)	5	200	1021.35	74.34	0.00	inside
species richness	disturbance+level+(1|site/cushion ID)	8	200	1024.84	77.83	0.00	inside

species richness	level+(1|site/cushion ID)	7	200	1028.22	81.21	0.00	inside
species richness	disturbance+(1|site/cushion ID)	5	200	1044.21	97.21	0.00	inside
species richness	(1|site/cushion ID)	4	200	1045.27	98.26	0.00	inside
shannon diversity	level+disturbance*area+cushion+(1|site/cushion ID)	11	198	342.07	0.00	0.33	inside
shannon diversity	disturbance+level+area+cushion+(1|site/cushion ID)	10	198	343.24	1.16	0.18	inside
shannon diversity	disturbance*area+cushion+(1|site/cushion ID)	8	198	343.57	1.50	0.16	inside
shannon diversity	cushion+area+(1|site/cushion ID)	6	198	344.75	2.68	0.09	inside
shannon diversity	disturbance+cushion+area+(1|site/cushion ID)	7	198	345.01	2.94	0.08	inside
shannon diversity	disturbance+level*area+cushion+(1|site/cushion ID)	13	198	345.44	3.36	0.06	inside
shannon diversity	cushion+area+level+(1|site/cushion ID)	9	198	345.64	3.57	0.06	inside
shannon diversity	cushion+area*level+(1|site/cushion ID)	12	198	347.89	5.82	0.02	inside
shannon diversity	disturbance*level+area+cushion+(1|site/cushion ID)	13	198	348.79	6.72	0.01	inside
shannon diversity	(1|site/cushion ID)	4	200	349.25	7.18	0.01	inside
shannon diversity	disturbance*cushion+area+(1|site/cushion ID)	8	198	349.63	7.56	0.01	inside
shannon diversity	level+(1|site/cushion ID)	7	200	350.54	8.47	0.00	inside
shannon diversity	disturbance+level+(1|site/cushion ID)	8	200	352.15	10.08	0.00	inside
shannon diversity	disturbance+(1|site/cushion ID)	5	200	352.37	10.29	0.00	inside
shannon diversity	cushion+(1|site/cushion ID)	5	200	354.80	12.72	0.00	inside
shannon diversity	cushion*level+area+(1|site/cushion ID)	12	198	354.89	12.82	0.00	inside
shannon diversity	cushion+level+(1|site/cushion ID)	8	200	356.15	14.08	0.00	inside
shannon diversity	disturbance+level+cushion+(1|site/cushion ID)	9	200	357.78	15.71	0.00	inside
shannon diversity	disturbance+cushion+(1|site/cushion ID)	6	200	357.93	15.86	0.00	inside
shannon diversity	disturbance*level+(1|site/cushion ID)	11	200	359.23	17.16	0.00	inside
shannon diversity	disturbance*level*area+cushion+(1|site/cushion ID)	20	198	359.69	17.61	0.00	inside
shannon diversity	level+disturbance*cushion+(1|site/cushion ID)	10	200	362.42	20.34	0.00	inside
shannon diversity	disturbance*cushion+(1|site/cushion ID)	7	200	362.50	20.42	0.00	inside
shannon diversity	disturbance*level+cushion+(1|site/cushion ID)	12	200	364.94	22.86	0.00	inside
shannon diversity	cushion*level+(1|site/cushion ID)	11	200	365.71	23.64	0.00	inside
shannon diversity	disturbance+level*cushion+(1|site/cushion ID)	12	200	367.42	25.34	0.00	inside

shannon diversity	disturbance*level*cushion+(1|site/cushion ID)	19	200	381.76	39.68	0.00	inside
vegetation cover	disturbance*level*area+cushion+(1|site/cushion ID)	20	198	1729.72	0.00	1.00	inside
vegetation cover	disturbance*level*cushion+(1|site/cushion ID)	19	200	1744.12	14.41	0.00	inside
vegetation cover	disturbance*level+area+cushion+(1|site/cushion ID)	13	198	1763.99	34.27	0.00	inside
vegetation cover	disturbance+level*area+cushion+(1|site/cushion ID)	13	198	1773.71	44.00	0.00	inside
vegetation cover	cushion*level+area+(1|site/cushion ID)	12	198	1781.20	51.48	0.00	inside
vegetation cover	level+disturbance*area+cushion+(1|site/cushion ID)	11	198	1781.56	51.84	0.00	inside
vegetation cover	cushion+area*level+(1|site/cushion ID)	12	198	1782.32	52.60	0.00	inside
vegetation cover	disturbance*level+cushion+(1|site/cushion ID)	12	200	1787.83	58.11	0.00	inside
vegetation cover	disturbance+level+area+cushion+(1|site/cushion ID)	10	198	1787.86	58.14	0.00	inside
vegetation cover	disturbance+level*cushion+(1|site/cushion ID)	12	200	1795.31	65.60	0.00	inside
vegetation cover	cushion+area+level+(1|site/cushion ID)	9	198	1796.57	66.85	0.00	inside
vegetation cover	cushion*level+(1|site/cushion ID)	11	200	1801.84	72.12	0.00	inside
vegetation cover	level+disturbance*cushion+(1|site/cushion ID)	10	200	1807.62	77.90	0.00	inside
vegetation cover	disturbance+level+cushion+(1|site/cushion ID)	9	200	1810.81	81.09	0.00	inside
vegetation cover	cushion+level+(1|site/cushion ID)	8	200	1817.40	87.68	0.00	inside
vegetation cover	disturbance*area+cushion+(1|site/cushion ID)	8	198	1824.57	94.85	0.00	inside
vegetation cover	disturbance*cushion+area+(1|site/cushion ID)	8	198	1827.35	97.64	0.00	inside
vegetation cover	disturbance+cushion+area+(1|site/cushion ID)	7	198	1830.60	100.88	0.00	inside
vegetation cover	cushion+area+(1|site/cushion ID)	6	198	1836.04	106.32	0.00	inside
vegetation cover	disturbance*cushion+(1|site/cushion ID)	7	200	1849.56	119.84	0.00	inside
vegetation cover	disturbance+cushion+(1|site/cushion ID)	6	200	1852.81	123.10	0.00	inside
vegetation cover	cushion+(1|site/cushion ID)	5	200	1856.98	127.26	0.00	inside
vegetation cover	disturbance*level+(1|site/cushion ID)	11	200	1895.86	166.15	0.00	inside
vegetation cover	disturbance+level+(1|site/cushion ID)	8	200	1916.83	187.11	0.00	inside
vegetation cover	level+(1|site/cushion ID)	7	200	1922.62	192.90	0.00	inside
vegetation cover	disturbance+(1|site/cushion ID)	5	200	1953.17	223.45	0.00	inside
vegetation cover	(1|site/cushion ID)	4	200	1957.29	227.58	0.00	inside

species richness	disturbance*level*area+cushion+(1|site/cushion ID)	20	198	972.41	0.00	0.79	neighboring
species richness	disturbance*level+area+cushion+(1|site/cushion ID)	13	198	975.13	2.72	0.20	neighboring
species richness	disturbance*level*cushion+(1|site/cushion ID)	19	200	981.45	9.04	0.01	neighboring
species richness	disturbance*level+(1|site/cushion ID)	11	200	984.86	12.45	0.00	neighboring
species richness	disturbance*level+cushion+(1|site/cushion ID)	12	200	985.59	13.18	0.00	neighboring
species richness	level+disturbance*area+cushion+(1|site/cushion ID)	11	198	986.74	14.33	0.00	neighboring
species richness	disturbance+level+area+cushion+(1|site/cushion ID)	10	198	986.79	14.38	0.00	neighboring
species richness	disturbance+level*area+cushion+(1|site/cushion ID)	13	198	987.71	15.30	0.00	neighboring
species richness	level+disturbance*cushion+(1|site/cushion ID)	10	200	994.20	21.79	0.00	neighboring
species richness	disturbance+level+(1|site/cushion ID)	8	200	995.76	23.35	0.00	neighboring
species richness	disturbance+level*cushion+(1|site/cushion ID)	12	200	995.80	23.39	0.00	neighboring
species richness	disturbance+level+cushion+(1|site/cushion ID)	9	200	996.42	24.01	0.00	neighboring
species richness	cushion*level+area+(1|site/cushion ID)	12	198	1005.93	33.52	0.00	neighboring
species richness	cushion+area*level+(1|site/cushion ID)	12	198	1006.31	33.90	0.00	neighboring
species richness	cushion+area+level+(1|site/cushion ID)	9	198	1006.78	34.37	0.00	neighboring
species richness	cushion*level+(1|site/cushion ID)	11	200	1012.52	40.11	0.00	neighboring
species richness	level+(1|site/cushion ID)	7	200	1012.57	40.16	0.00	neighboring
species richness	disturbance*cushion+area+(1|site/cushion ID)	8	198	1012.84	40.43	0.00	neighboring
species richness	cushion+level+(1|site/cushion ID)	8	200	1013.20	40.79	0.00	neighboring
species richness	disturbance*area+cushion+(1|site/cushion ID)	8	198	1014.50	42.09	0.00	neighboring
species richness	disturbance+cushion+area+(1|site/cushion ID)	7	198	1015.19	42.78	0.00	neighboring
species richness	disturbance*cushion+(1|site/cushion ID)	7	200	1023.56	51.15	0.00	neighboring
species richness	disturbance+(1|site/cushion ID)	5	200	1025.26	52.84	0.00	neighboring
species richness	disturbance+cushion+(1|site/cushion ID)	6	200	1025.85	53.44	0.00	neighboring
species richness	cushion+area+(1|site/cushion ID)	6	198	1026.92	54.51	0.00	neighboring
species richness	(1|site/cushion ID)	4	200	1033.99	61.58	0.00	neighboring
species richness	cushion+(1|site/cushion ID)	5	200	1034.56	62.15	0.00	neighboring
shannon diversity	disturbance+level+(1|site/cushion ID)	8	200	203.42	0.00	0.77	neighboring
shannon diversity	disturbance+level+cushion+(1|site/cushion ID)	9	200	206.91	3.49	0.13	neighboring

shannon diversity	disturbance*level+(1|site/cushion ID)	11	200	209.69	6.26	0.03	neighboring
shannon diversity	level+disturbance*cushion+(1|site/cushion ID)	10	200	210.67	7.25	0.02	neighboring
shannon diversity	disturbance+level+area+cushion+(1|site/cushion ID)	10	198	211.07	7.65	0.02	neighboring
shannon diversity	level+(1|site/cushion ID)	7	200	212.29	8.87	0.01	neighboring
shannon diversity	disturbance*level+cushion+(1|site/cushion ID)	12	200	213.24	9.82	0.01	neighboring
shannon diversity	disturbance+(1|site/cushion ID)	5	200	213.92	10.50	0.00	neighboring
shannon diversity	cushion+level+(1|site/cushion ID)	8	200	215.76	12.34	0.00	neighboring
shannon diversity	level+disturbance*area+cushion+(1|site/cushion ID)	11	198	216.07	12.65	0.00	neighboring
shannon diversity	disturbance*level+area+cushion+(1|site/cushion ID)	13	198	216.74	13.32	0.00	neighboring
shannon diversity	(1|site/cushion ID)	4	200	216.85	13.43	0.00	neighboring
shannon diversity	disturbance+cushion+(1|site/cushion ID)	6	200	217.34	13.92	0.00	neighboring
shannon diversity	disturbance+level*cushion+(1|site/cushion ID)	12	200	219.13	15.71	0.00	neighboring
shannon diversity	disturbance+cushion+area+(1|site/cushion ID)	7	198	220.09	16.67	0.00	neighboring
shannon diversity	cushion+(1|site/cushion ID)	5	200	220.24	16.82	0.00	neighboring
shannon diversity	disturbance*cushion+(1|site/cushion ID)	7	200	221.04	17.62	0.00	neighboring
shannon diversity	cushion+area+level+(1|site/cushion ID)	9	198	221.58	18.16	0.00	neighboring
shannon diversity	disturbance+level*area+cushion+(1|site/cushion ID)	13	198	222.54	19.11	0.00	neighboring
shannon diversity	disturbance*cushion+area+(1|site/cushion ID)	8	198	223.62	20.20	0.00	neighboring
shannon diversity	disturbance*area+cushion+(1|site/cushion ID)	8	198	224.42	21.00	0.00	neighboring
shannon diversity	cushion+area+(1|site/cushion ID)	6	198	224.90	21.48	0.00	neighboring
shannon diversity	cushion*level+(1|site/cushion ID)	11	200	227.91	24.49	0.00	neighboring
shannon diversity	cushion+area*level+(1|site/cushion ID)	12	198	231.69	28.27	0.00	neighboring
shannon diversity	cushion*level+area+(1|site/cushion ID)	12	198	233.44	30.02	0.00	neighboring
shannon diversity	disturbance*level*cushion+(1|site/cushion ID)	19	200	239.14	35.72	0.00	neighboring
shannon diversity	disturbance*level*area+cushion+(1|site/cushion ID)	20	198	243.52	40.10	0.00	neighboring
vegetation cover	disturbance*level*area+cushion+(1|site/cushion ID)	20	198	1670.06	0.00	1.00	neighboring
vegetation cover	disturbance*level*cushion+(1|site/cushion ID)	19	200	1689.52	19.46	0.00	neighboring
vegetation cover	disturbance*level+area+cushion+(1|site/cushion ID)	13	198	1698.80	28.74	0.00	neighboring
vegetation cover	disturbance+level*area+cushion+(1|site/cushion ID)	13	198	1716.65	46.59	0.00	neighboring

vegetation cover	disturbance*level+cushion+(1|site/cushion ID)	12	200	1717.66	47.60	0.00	neighboring
vegetation cover	disturbance*level+(1|site/cushion ID)	11	200	1718.74	48.68	0.00	neighboring
vegetation cover	level+disturbance*area+cushion+(1|site/cushion ID)	11	198	1724.56	54.50	0.00	neighboring
vegetation cover	disturbance+level+area+cushion+(1|site/cushion ID)	10	198	1727.29	57.23	0.00	neighboring
vegetation cover	cushion*level+area+(1|site/cushion ID)	12	198	1732.33	62.27	0.00	neighboring
vegetation cover	cushion+area*level+(1|site/cushion ID)	12	198	1732.95	62.89	0.00	neighboring
vegetation cover	disturbance+level*cushion+(1|site/cushion ID)	12	200	1735.79	65.73	0.00	neighboring
vegetation cover	level+disturbance*cushion+(1|site/cushion ID)	10	200	1743.92	73.86	0.00	neighboring
vegetation cover	cushion+area+level+(1|site/cushion ID)	9	198	1744.42	74.36	0.00	neighboring
vegetation cover	disturbance+level+cushion+(1|site/cushion ID)	9	200	1747.36	77.30	0.00	neighboring
vegetation cover	disturbance+level+(1|site/cushion ID)	8	200	1748.50	78.44	0.00	neighboring
vegetation cover	cushion*level+(1|site/cushion ID)	11	200	1754.92	84.86	0.00	neighboring
vegetation cover	cushion+level+(1|site/cushion ID)	8	200	1766.57	96.51	0.00	neighboring
vegetation cover	level+(1|site/cushion ID)	7	200	1767.73	97.67	0.00	neighboring
vegetation cover	disturbance*cushion+area+(1|site/cushion ID)	8	198	1769.14	99.08	0.00	neighboring
vegetation cover	disturbance*area+cushion+(1|site/cushion ID)	8	198	1769.70	99.64	0.00	neighboring
vegetation cover	disturbance+cushion+area+(1|site/cushion ID)	7	198	1772.80	102.74	0.00	neighboring
vegetation cover	cushion+area+(1|site/cushion ID)	6	198	1783.17	113.11	0.00	neighboring
vegetation cover	disturbance*cushion+(1|site/cushion ID)	7	200	1789.54	119.48	0.00	neighboring
vegetation cover	disturbance+cushion+(1|site/cushion ID)	6	200	1793.05	122.99	0.00	neighboring
vegetation cover	disturbance+(1|site/cushion ID)	5	200	1794.26	124.20	0.00	neighboring
vegetation cover	cushion+(1|site/cushion ID)	5	200	1804.31	134.25	0.00	neighboring
vegetation cover	(1|site/cushion ID)	4	200	1805.54	135.48	0.00	neighboring

B

**modeled parameter**	**model formula**	**k**	**n**	**AICc**	**Δ AICc**	**AICc weight**
species richness	disturbance*level*SWC*SOM+(1|site/cushion ID)	35	114	543.41	0.00	1.00
species richness	disturbance*level*SOM+(1|site/cushion ID)	19	114	586.99	43.58	0.00
species richness	disturbance*level*SOM+SWC+(1|site/cushion ID)	20	114	587.79	44.38	0.00
species richness	disturbance*level*SWC+(1|site/cushion ID)	19	114	588.13	44.72	0.00

species richness	disturbance*level*SWC+SOM+(1|site/cushion ID)	20	114	588.58	45.17	0.00
species richness	level*SOM*SWC+(1|site/cushion ID)	19	114	592.29	48.88	0.00
species richness	disturbance*level+(1|site/cushion ID)	11	114	594.19	50.78	0.00
species richness	disturbance*level+SOM+(1|site/cushion ID)	12	114	596.14	52.73	0.00
species richness	disturbance*level+SWC+(1|site/cushion ID)	12	114	596.19	52.78	0.00
species richness	disturbance*level+SOM+SWC+(1|site/cushion ID)	13	114	596.89	53.48	0.00
species richness	disturbance*level+SOM*SWC+(1|site/cushion ID)	14	114	598.47	55.06	0.00
species richness	disturbance+level+(1|site/cushion ID)	8	114	599.95	56.54	0.00
species richness	disturbance+level*SOM+(1|site/cushion ID)	12	114	601.24	57.83	0.00
species richness	disturbance+level+SWC+(1|site/cushion ID)	9	114	601.55	58.14	0.00
species richness	disturbance+level+SOM+(1|site/cushion ID)	9	114	601.62	58.20	0.00
species richness	level+disturbance*SOM+(1|site/cushion ID)	10	114	601.73	58.31	0.00
species richness	disturbance+level*SWC+(1|site/cushion ID)	12	114	601.87	58.46	0.00
species richness	disturbance+level*SOM+SWC+(1|site/cushion ID)	13	114	601.99	58.58	0.00
species richness	level+disturbance*SOM+SWC+(1|site/cushion ID)	11	114	602.03	58.61	0.00
species richness	level+disturbance*SWC+(1|site/cushion ID)	10	114	602.10	58.69	0.00
species richness	disturbance+level+SOM+SWC+(1|site/cushion ID)	10	114	602.17	58.76	0.00
species richness	disturbance+level*SWC+SOM+(1|site/cushion ID)	13	114	602.46	59.05	0.00
species richness	level+disturbance*SWC+SOM+(1|site/cushion ID)	11	114	602.50	59.09	0.00
species richness	level*SOM+(1|site/cushion ID)	11	114	602.63	59.22	0.00
species richness	level+(1|site/cushion ID)	7	114	602.96	59.54	0.00
species richness	level+SOM+(1|site/cushion ID)	8	114	603.31	59.90	0.00
species richness	level*SOM+SWC+(1|site/cushion ID)	12	114	603.48	60.07	0.00
species richness	level*SOM+SWC+(1|site/cushion ID)	12	114	603.48	60.07	0.00
species richness	level+SWC+(1|site/cushion ID)	8	114	603.58	60.17	0.00
species richness	disturbance+level+SOM*SWC+(1|site/cushion ID)	11	114	603.68	60.27	0.00
species richness	level+disturbance+SOM*SWC+(1|site/cushion ID)	11	114	603.68	60.27	0.00
species richness	level*SWC+(1|site/cushion ID)	11	114	603.77	60.35	0.00
species richness	level+SOM+SWC+(1|site/cushion ID)	9	114	604.15	60.73	0.00
species richness	disturbance*SOM+(1|site/cushion ID)	7	114	609.73	66.32	0.00

species richness	disturbance*SOM+SWC+(1|site/cushion ID)	8	114	609.78	66.36	0.00
species richness	disturbance*SOM+SWC*(1|site/cushion ID)	8	114	609.78	66.36	0.00
species richness	disturbance*SWC+(1|site/cushion ID)	7	114	609.91	66.50	0.00
species richness	disturbance+(1|site/cushion ID)	5	114	610.28	66.86	0.00
species richness	disturbance+SWC+(1|site/cushion ID)	6	114	610.32	66.90	0.00
species richness	disturbance+SOM+(1|site/cushion ID)	6	114	610.36	66.94	0.00
species richness	disturbance+SOM+SWC+(1|site/cushion ID)	7	114	610.83	67.42	0.00
species richness	SOM+(1|site/cushion ID)	5	114	610.91	67.50	0.00
species richness	SWC+(1|site/cushion ID)	5	114	611.26	67.85	0.00
species richness	SOM+SWC+(1|site/cushion ID)	6	114	611.54	68.13	0.00
species richness	(1|site/cushion ID)	4	114	612.21	68.80	0.00
species richness	SOM*SWC+(1|site/cushion ID)	7	114	612.83	69.41	0.00
shannon diversity	(1|site/cushion ID)	4	114	217.75	0.00	0.34
shannon diversity	SOM+(1|site/cushion ID)	5	114	219.10	1.36	0.17
shannon diversity	disturbance+(1|site/cushion ID)	5	114	219.34	1.59	0.15
shannon diversity	SWC+(1|site/cushion ID)	5	114	220.11	2.36	0.10
shannon diversity	level+(1|site/cushion ID)	7	114	221.58	3.83	0.05
shannon diversity	disturbance+SOM+(1|site/cushion ID)	6	114	222.34	4.59	0.03
shannon diversity	disturbance+level+(1|site/cushion ID)	8	114	222.40	4.66	0.03
shannon diversity	disturbance+SWC+(1|site/cushion ID)	6	114	222.87	5.12	0.03
shannon diversity	SOM+SWC+(1|site/cushion ID)	6	114	223.32	5.58	0.02
shannon diversity	level+SOM+(1|site/cushion ID)	8	114	224.23	6.48	0.01
shannon diversity	disturbance*SOM+(1|site/cushion ID)	7	114	224.65	6.91	0.01
shannon diversity	level+SWC+(1|site/cushion ID)	8	114	225.29	7.54	0.01
shannon diversity	disturbance*SWC+(1|site/cushion ID)	7	114	225.80	8.05	0.01
shannon diversity	disturbance+SOM+SWC+(1|site/cushion ID)	7	114	226.55	8.80	0.00
shannon diversity	disturbance+level+SOM+(1|site/cushion ID)	9	114	226.81	9.07	0.00
shannon diversity	disturbance+level+SWC+(1|site/cushion ID)	9	114	227.23	9.49	0.00
shannon diversity	SOM*SWC+(1|site/cushion ID)	7	114	228.04	10.29	0.00

shannon diversity	disturbance*SOM+SWC+(1|site/cushion ID)	8	114	228.53	10.78	0.00
shannon diversity	disturbance*SOM+SWC*(1|site/cushion ID)	8	114	228.53	10.78	0.00
shannon diversity	level+SOM+SWC+(1|site/cushion ID)	9	114	228.54	10.79	0.00
shannon diversity	disturbance*level+(1|site/cushion ID)	11	114	228.64	10.90	0.00
shannon diversity	level+disturbance*SOM+(1|site/cushion ID)	10	114	230.02	12.28	0.00
shannon diversity	level+disturbance*SWC+(1|site/cushion ID)	10	114	231.08	13.33	0.00
shannon diversity	disturbance+level+SOM+SWC+(1|site/cushion ID)	10	114	231.19	13.44	0.00
shannon diversity	disturbance*level+SOM+(1|site/cushion ID)	12	114	233.36	15.61	0.00
shannon diversity	disturbance*level+SWC+(1|site/cushion ID)	12	114	233.74	16.00	0.00
shannon diversity	level+disturbance*SOM+SWC+(1|site/cushion ID)	11	114	234.23	16.48	0.00
shannon diversity	level*SOM+(1|site/cushion ID)	11	114	234.48	16.73	0.00
shannon diversity	level+disturbance*SWC+SOM+(1|site/cushion ID)	11	114	235.05	17.30	0.00
shannon diversity	disturbance+level+SOM*SWC+(1|site/cushion ID)	11	114	235.91	18.17	0.00
shannon diversity	level+disturbance+SOM*SWC+(1|site/cushion ID)	11	114	235.91	18.17	0.00
shannon diversity	level*SWC+(1|site/cushion ID)	11	114	236.64	18.89	0.00
shannon diversity	disturbance+level*SOM+(1|site/cushion ID)	12	114	236.86	19.12	0.00
shannon diversity	disturbance*level+SOM+SWC+(1|site/cushion ID)	13	114	237.77	20.02	0.00
shannon diversity	disturbance+level*SWC+(1|site/cushion ID)	12	114	238.40	20.65	0.00
shannon diversity	level*SOM+SWC+(1|site/cushion ID)	12	114	238.75	21.00	0.00
shannon diversity	level*SOM+SWC+(1|site/cushion ID)	12	114	238.75	21.00	0.00
shannon diversity	disturbance+level*SOM+SWC+(1|site/cushion ID)	13	114	241.21	23.46	0.00
shannon diversity	disturbance+level*SWC+SOM+(1|site/cushion ID)	13	114	242.42	24.68	0.00
shannon diversity	disturbance*level+SOM*SWC+(1|site/cushion ID)	14	114	242.54	24.80	0.00
shannon diversity	disturbance*level*SOM+(1|site/cushion ID)	19	114	248.72	30.98	0.00
shannon diversity	level*SOM*SWC+(1|site/cushion ID)	19	114	251.50	33.76	0.00
shannon diversity	disturbance*level*SWC+(1|site/cushion ID)	19	114	252.55	34.81	0.00
shannon diversity	disturbance*level*SOM+SWC+(1|site/cushion ID)	20	114	252.98	35.24	0.00
shannon diversity	disturbance*level*SWC+SOM+(1|site/cushion ID)	20	114	256.57	38.83	0.00
shannon diversity	disturbance*level*SWC*SOM+(1|site/cushion ID)	35	114	264.46	46.72	0.00

vegetation cover	disturbance*level*SWC*SOM+(1|site/cushion ID)	35	114	912.47	0.00	1.00
vegetation cover	disturbance*level*SOM+SWC+(1|site/cushion ID)	20	114	1016.11	103.64	0.00
vegetation cover	disturbance*level*SOM+(1|site/cushion ID)	19	114	1019.68	107.21	0.00
vegetation cover	disturbance*level*SWC+SOM+(1|site/cushion ID)	20	114	1019.71	107.24	0.00
vegetation cover	level*SOM*SWC+(1|site/cushion ID)	19	114	1022.08	109.61	0.00
vegetation cover	disturbance*level*SWC+(1|site/cushion ID)	19	114	1023.33	110.86	0.00
vegetation cover	disturbance*level+SOM*SWC+(1|site/cushion ID)	14	114	1053.92	141.45	0.00
vegetation cover	disturbance*level+SOM+SWC+(1|site/cushion ID)	13	114	1056.61	144.14	0.00
vegetation cover	disturbance+level*SOM+SWC+(1|site/cushion ID)	13	114	1058.94	146.47	0.00
vegetation cover	disturbance*level+SWC+(1|site/cushion ID)	12	114	1060.18	147.71	0.00
vegetation cover	disturbance+level*SWC+SOM+(1|site/cushion ID)	13	114	1061.40	148.93	0.00
vegetation cover	disturbance*level+SOM+(1|site/cushion ID)	12	114	1062.08	149.61	0.00
vegetation cover	level*SOM+SWC+(1|site/cushion ID)	12	114	1063.37	150.90	0.00
vegetation cover	level*SOM+SWC+(1|site/cushion ID)	12	114	1063.37	150.90	0.00
vegetation cover	disturbance+level*SOM+(1|site/cushion ID)	12	114	1063.49	151.02	0.00
vegetation cover	disturbance+level*SWC+(1|site/cushion ID)	12	114	1064.92	152.45	0.00
vegetation cover	disturbance*level+(1|site/cushion ID)	11	114	1066.02	153.55	0.00
vegetation cover	level*SOM+(1|site/cushion ID)	11	114	1067.60	155.13	0.00
vegetation cover	level*SWC+(1|site/cushion ID)	11	114	1069.03	156.56	0.00
vegetation cover	level+disturbance*SOM+SWC+(1|site/cushion ID)	11	114	1069.21	156.74	0.00
vegetation cover	level+disturbance*SWC+SOM+(1|site/cushion ID)	11	114	1069.62	157.15	0.00
vegetation cover	disturbance+level+SOM*SWC+(1|site/cushion ID)	11	114	1070.44	157.97	0.00
vegetation cover	level+disturbance+SOM*SWC+(1|site/cushion ID)	11	114	1070.44	157.97	0.00
vegetation cover	disturbance+level+SOM+SWC+(1|site/cushion ID)	10	114	1073.25	160.79	0.00
vegetation cover	level+disturbance*SWC+(1|site/cushion ID)	10	114	1073.46	160.99	0.00
vegetation cover	level+disturbance*SOM+(1|site/cushion ID)	10	114	1075.44	162.97	0.00
vegetation cover	disturbance+level+SWC+(1|site/cushion ID)	9	114	1077.16	164.69	0.00
vegetation cover	level+SOM+SWC+(1|site/cushion ID)	9	114	1078.98	166.51	0.00
vegetation cover	disturbance+level+SOM+(1|site/cushion ID)	9	114	1080.35	167.88	0.00
vegetation cover	level+SWC+(1|site/cushion ID)	8	114	1082.20	169.73	0.00

vegetation cover	level+SOM+(1|site/cushion ID)	8	114	1084.85	172.38	0.00
vegetation cover	disturbance+level+(1|site/cushion ID)	8	114	1086.70	174.23	0.00
vegetation cover	disturbance*SOM+SWC+(1|site/cushion ID)	8	114	1090.08	177.61	0.00
vegetation cover	disturbance*SOM+SWC*(1|site/cushion ID)	8	114	1090.08	177.61	0.00
vegetation cover	disturbance*SWC+(1|site/cushion ID)	7	114	1093.61	181.14	0.00
vegetation cover	SOM*SWC+(1|site/cushion ID)	7	114	1093.79	181.33	0.00
vegetation cover	disturbance+SOM+SWC+(1|site/cushion ID)	7	114	1094.12	181.65	0.00
vegetation cover	level+(1|site/cushion ID)	7	114	1095.30	182.83	0.00
vegetation cover	disturbance*SOM+(1|site/cushion ID)	7	114	1097.09	184.62	0.00
vegetation cover	SOM+SWC+(1|site/cushion ID)	6	114	1097.60	185.13	0.00
vegetation cover	disturbance+SWC+(1|site/cushion ID)	6	114	1097.68	185.21	0.00
vegetation cover	disturbance+SOM+(1|site/cushion ID)	6	114	1100.98	188.51	0.00
vegetation cover	SWC+(1|site/cushion ID)	5	114	1101.23	188.76	0.00
vegetation cover	SOM+(1|site/cushion ID)	5	114	1104.29	191.82	0.00
vegetation cover	disturbance+(1|site/cushion ID)	5	114	1116.87	204.40	0.00
vegetation cover	(1|site/cushion ID)	4	114	1123.21	210.74	0.00

Table B3. Full list of Linear Mixed Models to test how species community competitiveness is influenced by disturbance, elevation, and cushion presence. Black bars differentiate model sets, k = number of parameters, n = sample size.

modeled parameter	model formula	k	n	AICc	Δ AICc	AICc weight	species
competition	level+(1|site)	6	198	-20.07	0.00	0.53	inside
competition	cushion+level+(1|site)	7	198	-19.73	0.34	0.44	inside
competition	disturbance+level+(1|site)	7	198	-12.81	7.26	0.01	inside
competition	disturbance+level+cushion+(1|site)	8	198	-12.42	7.65	0.01	inside
competition	disturbance*level+(1|site)	10	198	-7.87	12.19	0.00	inside
competition	disturbance*level+cushion+(1|site)	11	198	-7.67	12.39	0.00	inside
competition	level+disturbance*cushion+(1|site)	9	198	-6.50	13.57	0.00	inside
competition	cushion*level+(1|site)	10	198	-4.95	15.12	0.00	inside
competition	disturbance+level*cushion+(1|site)	11	198	2.42	22.49	0.00	inside
competition	disturbance*level*cushion+(1|site)	18	198	25.53	45.60	0.00	inside
competition	(1|site)	3	198	51.12	71.19	0.00	inside
competition	cushion+(1|site)	4	198	53.12	73.19	0.00	inside
competition	disturbance+(1|site)	4	198	56.95	77.02	0.00	inside
competition	cushion+disturbance+(1|site)	5	198	59.01	79.08	0.00	inside
competition	cushion*disturbance+(1|site)	6	198	64.40	84.46	0.00	inside
competition	level+(1|site)	6	200	-122.69	0.00	0.50	neighboring
competition	cushion+level+(1|site)	7	200	-122.57	0.12	0.47	neighboring
competition	disturbance+level+(1|site)	7	200	-115.09	7.60	0.01	neighboring
competition	disturbance+level+cushion+(1|site)	8	200	-114.92	7.78	0.01	neighboring
competition	disturbance*level+(1|site)	10	200	-109.58	13.11	0.00	neighboring
competition	disturbance*level+cushion+(1|site)	9	200	-108.69	14.00	0.00	neighboring
competition	level+disturbance*cushion+(1|site)	11	200	-101.85	20.84	0.00	neighboring
competition	cushion*level+(1|site)	10	200	-98.32	24.37	0.00	neighboring
competition	disturbance+level*cushion+(1|site)	11	200	-98.02	24.67	0.00	neighboring
competition	disturbance*level*cushion+(1|site)	18	200	-66.55	56.14	0.00	neighboring

competition	(1|site)	3	200	0.51	123.20	0.00	neighboring
competition	cushion+(1|site)	4	200	7.29	129.98	0.00	neighboring
competition	disturbance+(1|site)	4	200	7.46	130.15	0.00	neighboring
competition	cushion+disturbance+(1|site)	5	200	14.26	136.95	0.00	neighboring
competition	cushion*disturbance+(1|site)	6	200	19.83	142.52	0.00	neighboring

Table B4. Linear Mixed Models used to test the effects of disturbance and elevation on soil parameters. Black bars differentiate model sets, and k = number of parameters, n = sample size.

modeled parameter	model formula	k	n	AICc	Δ AICc	AICc weight
SOM	disturbance*level*cushion+(1|site/cushion ID)	19	114	810.86	0.00	1.00
SOM	disturbance*level+cushion+(1|site/cushion ID)	12	114	836.90	26.04	0.00
SOM	disturbance*level+(1|site/cushion ID)	11	114	837.83	26.97	0.00
SOM	disturbance+level*cushion+(1|site/cushion ID)	12	114	856.43	45.57	0.00
SOM	level+disturbance*cushion+(1|site/cushion ID)	10	114	860.92	50.06	0.00
SOM	disturbance+level+cushion+(1|site/cushion ID)	9	114	863.61	52.75	0.00
SOM	disturbance+level+(1|site/cushion ID)	8	114	864.69	53.83	0.00
SOM	cushion*level+(1|site/cushion ID)	11	114	878.42	67.56	0.00
SOM	cushion+level+(1|site/cushion ID)	8	114	885.74	74.88	0.00
SOM	level+(1|site/cushion ID)	7	114	886.86	76.00	0.00
SOM	cushion*disturbance+(1|site/cushion ID)	7	114	892.96	82.10	0.00
SOM	cushion+disturbance+(1|site/cushion ID)	6	114	895.79	84.94	0.00
SOM	disturbance+(1|site/cushion ID)	5	114	897.00	86.14	0.00
SOM	cushion+(1|site/cushion ID)	5	114	910.54	99.68	0.00
SOM	(1|site/cushion ID)	4	114	911.79	100.93	0.00
SWC	disturbance*level*cushion+(1|site/cushion ID)	19	114	801.49	0.00	1.00
SWC	disturbance*level+cushion+(1|site/cushion ID)	12	114	828.54	27.06	0.00
SWC	disturbance*level+(1|site/cushion ID)	11	114	829.27	27.79	0.00
SWC	disturbance+level*cushion+(1|site/cushion ID)	12	114	854.76	53.27	0.00
SWC	level+disturbance*cushion+(1|site/cushion ID)	10	114	858.32	56.84	0.00
SWC	disturbance+level+cushion+(1|site/cushion ID)	9	114	862.16	60.67	0.00
SWC	disturbance+level+(1|site/cushion ID)	8	114	863.03	61.54	0.00
SWC	cushion*level+(1|site/cushion ID)	11	114	865.62	64.13	0.00
SWC	cushion+level+(1|site/cushion ID)	8	114	873.16	71.67	0.00
SWC	level+(1|site/cushion ID)	7	114	874.07	72.59	0.00

SWC	cushion*disturbance+(1|site/cushion ID)	7	114	882.33	80.84	0.00
SWC	cushion+disturbance+(1|site/cushion ID)	6	114	886.30	84.81	0.00
SWC	disturbance+(1|site/cushion ID)	5	114	887.30	85.81	0.00
SWC	cushion+(1|site/cushion ID)	5	114	894.26	92.77	0.00
SWC	(1|site/cushion ID)	4	114	895.30	93.81	0.00

Table B5. Full list of Linear Mixed Models testing the effects of disturbance and elevational level on Relative Interaction Indices (RII) and Bray-Curtis Dissimilarity indices. Black bars differentiate model sets, and k = number of parameters, n = sample size.

modeled parameter	model formula	k	n	AICc	Δ AICc	AICc weight	species
RII: species richness	(1|site)	3	100	39.41	0.00	0.95	inside
RII: species richness	disturbance+(1|site)	4	100	45.53	6.11	0.04	inside
RII: species richness	level+(1|site)	6	100	50.35	10.93	0.00	inside
RII: species richness	disturbance+level+(1|site)	7	100	56.53	17.12	0.00	inside
RII: species richness	disturbance*level+(1|site)	10	100	67.05	27.63	0.00	inside
RII: shannon diversity	(1|site)	3	100	103.55	0.00	0.93	inside
RII: shannon diversity	disturbance+(1|site)	4	100	108.92	5.38	0.06	inside
RII: shannon diversity	level+(1|site)	6	100	114.79	11.24	0.00	inside
RII: shannon diversity	disturbance+level+(1|site)	7	100	120.36	16.82	0.00	inside
RII: shannon diversity	disturbance*level+(1|site)	10	100	127.72	24.18	0.00	inside
RII: % vegetation cover	(1|site)	3	100	86.07	0.00	0.94	inside
RII: % vegetation cover	disturbance+(1|site)	4	100	91.70	5.63	0.06	inside
RII: % vegetation cover	level+(1|site)	6	100	97.77	11.70	0.00	inside
RII: % vegetation cover	disturbance+level+(1|site)	7	100	103.51	17.44	0.00	inside
RII: % vegetation cover	disturbance*level+(1|site)	10	100	111.23	25.16	0.00	inside
RII: species richness	(1|site)	3	100	-82.52	0.00	0.95	neighboring
RII: species richness	disturbance+(1|site)	4	100	-76.55	5.97	0.05	neighboring
RII: species richness	level+(1|site)	6	100	-63.94	18.58	0.00	neighboring
RII: species richness	disturbance+level+(1|site)	7	100	-57.71	24.81	0.00	neighboring
RII: species richness	disturbance*level+(1|site)	10	100	-44.13	38.39	0.00	neighboring
RII: shannon diversity	(1|site)	3	100	-110.56	0.00	0.96	neighboring
RII: shannon diversity	disturbance+(1|site)	4	100	-103.98	6.58	0.04	neighboring

RII: shannon diversity	level+(1|site)	6	100	-92.41	18.16	0.00	neighboring
RII: shannon diversity	disturbance+level+(1|site)	7	100	-85.63	24.93	0.00	neighboring
RII: shannon diversity	disturbance*level+(1|site)	10	100	-70.24	40.32	0.00	neighboring
RII: % vegetation cover	(1|site)	3	100	-15.11	0.00	0.97	neighboring
RII: % vegetation cover	disturbance+(1|site)	4	100	-8.45	6.66	0.03	neighboring
RII: % vegetation cover	level+(1|site)	6	100	1.06	16.17	0.00	neighboring
RII: % vegetation cover	disturbance+level+(1|site)	7	100	7.83	22.94	0.00	neighboring
RII: % vegetation cover	disturbance*level+(1|site)	10	100	17.85	32.96	0.00	neighboring

Table B6. Predicted values modeled using the parameter estimates and simulated data (shown in table) from the most parsimonious models for inside species vegetation cover, neighboring species vegetation cover, and neighboring species richness, which all include a three-way interaction between disturbance, cushion area, and level. Differences between disturbed and undisturbed predicted values suggest that disturbance effects are strongest at middle elevations (levels 2 and 3). Black bars differentiate models.

predicted parameter	species	disturbance	cushion area	level	cushion presence	prediction	difference = disturbed - undisturbed
vegetation cover	inside	0	-0.58	1	1	23.11	
vegetation cover	inside	1	-0.58	1	1	25.24	2.13
vegetation cover	inside	0	0.19	1	1	25.50	
vegetation cover	inside	1	0.19	1	1	25.83	0.33
vegetation cover	inside	0	-0.58	2	1	40.91	
vegetation cover	inside	1	-0.58	2	1	23.00	-17.91
vegetation cover	inside	0	0.19	2	1	50.65	
vegetation cover	inside	1	0.19	2	1	32.55	-18.10
vegetation cover	inside	0	-0.58	3	1	30.65	
vegetation cover	inside	1	-0.58	3	1	14.65	-15.99
vegetation cover	inside	0	0.19	3	1	40.99	
vegetation cover	inside	1	0.19	3	1	15.72	-25.26
vegetation cover	inside	0	-0.58	4	1	-5.42	
vegetation cover	inside	1	-0.58	4	1	6.76	12.18
vegetation cover	inside	0	0.19	4	1	4.20	
vegetation cover	inside	1	0.19	4	1	17.40	13.20
vegetation cover	neighboring	0	-0.76	1	1	75.91	
vegetation cover	neighboring	1	-0.76	1	1	68.29	-7.62
vegetation cover	neighboring	0	0.51	1	1	55.57	
vegetation cover	neighboring	1	0.51	1	1	63.88	8.31
vegetation cover	neighboring	0	-0.76	2	1	88.87	
vegetation cover	neighboring	1	-0.76	2	1	53.90	-34.97
vegetation cover	neighboring	0	0.51	2	1	90.47	

vegetation cover	neighboring	1	0.51	2	1	56.40	-34.08
vegetation cover	neighboring	0	-0.76	3	1	73.62	
vegetation cover	neighboring	1	-0.76	3	1	51.07	-22.55
vegetation cover	neighboring	0	0.51	3	1	75.33	
vegetation cover	neighboring	1	0.51	3	1	45.64	-29.69
vegetation cover	neighboring	0	-0.76	4	1	30.32	
vegetation cover	neighboring	1	-0.76	4	1	39.08	8.76
vegetation cover	neighboring	0	0.51	4	1	30.20	
vegetation cover	neighboring	1	0.51	4	1	36.69	6.49
species richness	neighboring	0	-0.76	1	1	11.61	
species richness	neighboring	1	-0.76	1	1	10.48	-1.14
species richness	neighboring	0	0.51	1	1	11.60	
species richness	neighboring	1	0.51	1	1	11.17	-0.43
species richness	neighboring	0	-0.76	2	1	12.93	
species richness	neighboring	1	-0.76	2	1	8.84	-4.09
species richness	neighboring	0	0.51	2	1	14.60	
species richness	neighboring	1	0.51	2	1	9.97	-4.63
species richness	neighboring	0	-0.76	3	1	10.57	
species richness	neighboring	1	-0.76	3	1	7.44	-3.13
species richness	neighboring	0	0.51	3	1	13.04	
species richness	neighboring	1	0.51	3	1	7.35	-5.69
species richness	neighboring	0	-0.76	4	1	6.97	
species richness	neighboring	1	-0.76	4	1	5.71	-1.26
species richness	neighboring	0	0.51	4	1	7.66	
species richness	neighboring	1	0.51	4	1	8.15	0.49
